# Genome-wide profiling identifies the THYT1 signature as a distinctive feature of widely metastatic Papillary Thyroid Carcinomas

**DOI:** 10.18632/oncotarget.22805

**Published:** 2017-12-01

**Authors:** Greta Gandolfi, Moira Ragazzi, Dario de Biase, Michela Visani, Eleonora Zanetti, Federica Torricelli, Valentina Sancisi, Mila Gugnoni, Gloria Manzotti, Luca Braglia, Silvio Cavuto, Domenico Franco Merlo, Giovanni Tallini, Andrea Frasoldati, Simonetta Piana, Alessia Ciarrocchi

**Affiliations:** ^1^ Laboratory of Translational Research, Azienda Unità Sanitaria Locale di Reggio Emilia - IRCCS, Reggio Emilia 42123, Italy; ^2^ Pathology Unit, Department of Oncology, Azienda Unità Sanitaria Locale di Reggio Emilia - IRCCS, Reggio Emilia 42123, Italy; ^3^ Department of Pharmacology and Biotechnology (FaBiT), University of Bologna, 40139 Bologna, Italy; ^4^ Department of Medicine, Dipartimento di Medicina Specialistica, Diagnostica e Sperimentale-DIMES, Anatomic Pathology Unit, Bellaria Hospital, University of Bologna, 40139 Bologna, Italy; ^5^ Research and Statistics Unit, Azienda Unità Sanitaria Locale di Reggio Emilia-IRCCS, Reggio Emilia, 42123, Italy; ^6^ Endocrinology Unit, Azienda Azienda Unitaria Sanitaria Locale di Reggio Emilia , Reggio Emilia 42123, Italy

**Keywords:** papillary thyroid carcinomas, distant metastases, copy number alterations, next generation sequencing, TERT duplication

## Abstract

**Background:**

Papillary Thyroid Carcinomas (PTCs) are generally indolent tumors. However, a small but significant percentage of PTCs behaves aggressively, progressing to a diffuse metastatic spreading and leading to patient's death. The lack of reliable markers for predicting the metastatic behavior of these tumors prevents a correct risk based stratification of the disease, thus contributing to the issue of patients’ overtreatment. In this study we aimed at identifying genetic features associated with the development of distant metastasis in PTCs.

**Results:**

We showed that DM PTCs are characterized by a moderate degree of copy number alterations but display low level of microsatellite instability and a low mutational burden. We identified duplication of Chr1q, duplication of Chr5p harboring the TERT genomic locus and mutations of TERT promoter as distinctive features of DM PTCs. These three genetic variables defined a signature (THYT1) that was significantly associated with a metastatic behavior and a shortened survival. We analyzed the THYT1 signature in PTCs fine needle aspirate biopsies (FNAB) and we demonstrating the applicability of this signature as a molecular marker in the pre-operative diagnostic setting of PTCs.

**Materials and Methods:**

A consecutive series of 2,937 thyroid malignancies, diagnosed at the Arcispedale S. Maria Nuova - IRCCS, Italy between 1978 and 2015 were searched to retrieve those who developed distant metastasis (DM, n = 50). We performed a deep profiling to explore the genomic landscape of these tumors.

**Conclusions:**

Overall our data identify the first genetic signature that independently predicts metastasis and negative outcome of PTCs, and lay the basis for the possible application of the THYT1 as prognostic marker to improve risk-based stratification and management of PTC patients.

## INTRODUCTION

Incidence of well-differentiated Papillary Thyroid Carcinoma (PTC) has steeply increased in the last decades worldwide [1–3]. PTCs are considered indolent tumors, with slow rate of growth, low metastatic potential and excellent prognosis [2, 4]. Nevertheless, a small percentage of differentiated PTCs behaves aggressively progressing to a diffuse metastatic spreading leading to death in 1–2% of total PTC patients [5].

Currently, it is not possible to distinguish at the time of diagnosis these highly aggressive PTCs from the rest of non-aggressive lesions since there is not a genetic or molecular signature available to predict the clinical progression of these tumors. Therefore, the issue of early accurate risk-based stratification in PTC is of particular relevance. Clinical management of patients with newly diagnosed PTCs may encompass a broad spectrum of options, ranging from sonographic surveillance to total thyroidectomy plus lymph node neck dissection and subsequent radioiodine ablation (RAI). The lack of effective criteria for risk stratification together with the increase in the number of newly diagnosed PTCs reported worldwide raise the chances of patient overtreatment.

The clinical feature majorly associated with the negative outcome of differentiated PTCs, is the presence of distant metastasis. PTCs that develop distant metastasis (DM) are rare and represent about 2–5% of all PTCs [4, 6, 7]. The genetic asset of these tumors is still largely unexplored.

In this work, we analyze the genomic landscape of a large retrospective series of differentiated PTCs with the intent of characterizing genetic features associated with the metastatic behavior of PTCs and the potential associations with overall survival (OS). We identified a signature of three genetic variables that we named THYT1. The presence of just one of these three genetic alterations was strongly associated with the development of distant metastasis. We explored the correlation of the THYT1 signature with patient survival probability. We also evaluated the feasibility of applying THYT1 to fine needle aspirate biopsies (FNAB) to predict aggressiveness of PTCs before surgery.

## RESULTS

### PTCs show low mutational load and negligible microsatellite instability (MSI)

**Figure 1:**
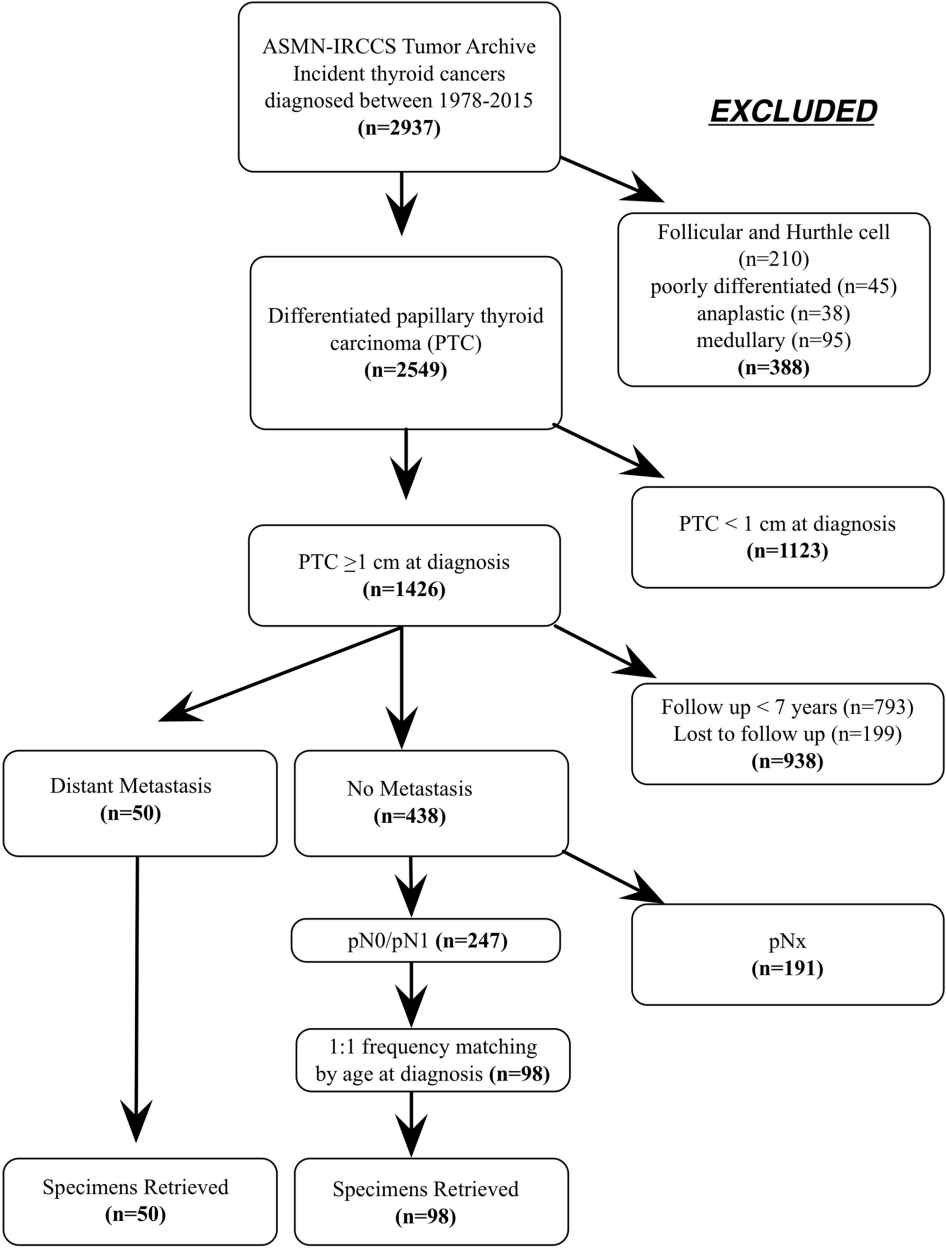
(**A**) Diagram of the study design and patients selection.

Clinical and pathological features of DM (*n* = 50) and Controls (*n* = 98) used in this study (Figure 1) are reported in Table 1. Of DM, 22 cases (44%) developed metastasis after the initial diagnosis (M0, metachronous metastases) and 28 patients (56%) had distant metastases at presentation (M1, synchronous metastases). Seven patients (14%) did not presented lymph nodal metastasis (pN0), 11 (22%) were pN1a and 32 (64%) were pN1b.

**Table 1:** Clinical features of DM and control patients included in this study

	DMs(*N* = 50)	Controls(*N* = 98)
Clinico-pathological features	N (%)	N (%)
Age at diagnosis, years (mean, 95% CI)	55.8 (50.6, 61.0)	53.6 (50.8, 56.4)
Gender		
Females	32 (64)	72 (73)
Males	18 (36)	26 (27)
Histological Diagnosis^a^		
C-PTC	25 (50)	71 (72)
TCV-PTC^*^	15 (30)	11 (11)
FV-PTC	8 (16)	15 (15)
ST-PTC	2 (4)	1 (1)
Stage (AJCC)		
I	2 (4)	44 (45)
II	9 (18)	5 (5)
III	8 (16)	33 (34)
IV	31 (62)	16 (16)
Follow Up Status		
Alive	20 (40)	90 (91.8)
Deceased	29 (58)	8 (8.2)
Lost to follow up	1 (2)	0 (0)
Follow Up, months^b^	117.2 (40.5–193.2)	143.4 (114.3–177.5)

^a^ C-PTC, classic PTC; TCV-PTC, tall cell variant PTC; FV-PTC, follicular variant PTC; ST-PTC, solid trabecular PTC;

^b^median, (25th and 75th centiles)

^*^TCV-PTC were defined as lesions that contained more than 50% of tall cells according with WHO guidelines

The genomic profile of DM and Control PTCs was assessed in 49 DM and 97 Control patients with good quality and quantity DNA samples available (Table 2). First, we evaluated the overall mutational profile of DM and Control PTCs, in a panel of 172 amplicons within 26 genes by Next Generation Sequencing (NGS). We also analyzed the mutation status of BRAF V600 and *TERT* promoter by direct sequencing and the presence of mutations in the *EIF1AX* by a NGS custom panel [8, 9]. Deep sequencing analysis of the 26 genes panel (Supplementary Table 1) in 21 DMs and 24 Controls revealed an overall low mutational load in PTCs. We detected 20 mutations in 12 genes, of which 12 mutations lead to amino acid substitution (Table 2, Supplementary Table 2). The only recurrent mutation was the BRAF V600E substitution, while the other mutations were private and found in only one sample (Table 2). No significant differences in the mutation frequencies and in the number of mutated genes were detected between DMs and Controls (Table 2).

**Table 2:** Genetic features by direct sequencing, targeted NGS and microsatellite instability (MSI) analysis

Direct Sequencing	DMs		Controls		
	No.^a^	Positive No (%)	No.	Positive No (%)	*p* value
**BRAF**					
*BRAF V600E*	48	17 (35.4)	84	46 (55)	0.05
***TERT promoter***	44		82		
*TERT prom C228T*		12 (27.3)		1 (1.2)	< 0.0001
*TERT prom C250T*		3 (6.8)		5 (6.1)	1
*TERT promoter any*		15 (34.0)		6 (7.3)	0.0002
***26 Genes TS panel^b^***	21		24		
*Total missense mutations*		7 (33.3)		4 (16.7)	
*Total mutated sample*		7 (33.3)		3 (12.5)	0.15
***EIF1AX exon 1-2c***	49		97		
*Total missense mutations*		4 (8.5)		5 (5.2)	
*Total mutated sample*		4 (8.5)		4 (4.1)	0.44
***MSI***	40		92		
*MSS*		40 (100)		89 (96.7)	
*MSI-L*		0 (0)		2 (2.2)	
*MSI-H*		0 (0)		1 (1.1)	

^a^Number of samples analyzed for each test. Differences are determined by the availability/ quality of DNA for each test.

^b^Genes included in the panel are listed in Supplementary Table 1. *BRAF* was included in the 26Genes panel the results are included in the direct sequencing results, since all the samples analyzed with both methods were concordant.

^c^Detailed list of mutations found in the 26 genes panel and in EIF1AX is reported in Supplementary Table 3.

The BRAF V600E mutation was found in 48% of all cases, and it tended to be more frequent in the Controls (55% of cases) than in the DMs (35%, *P* = 0.05) in accordance with previous reports [7, 9, 10]. *TERT* promoter was mutated in 16% of overall cases, and *TERT* promoter mutations (C228T and C250T) were significantly more frequent in DMs (34%) than in Controls (7%, *P* < 0.001) in accordance with our previous studies [11, 12] (Table 2).

Deep sequencing of *EIF1AX* detected missense mutations in 6.1% of the analyzed samples with not significant differences between DMs (8.2%) and Controls (4.1%) (*P* = 0.44) (Supplementary Table 2). We also evaluated the levels of microsatellite instability (MSI) in the two sets of samples (Table 2), observing an extremely high stability, without differences between the two groups (Table 2).

Overall, this analysis confirmed that PTCs are characterized by a low mutational load and a low degree of MSI and that this features do not discriminate between DMs and non-metastatic control lesions.

### Genome-wide copy number alteration (CNA) in DM PTCs and control PTCs

We used a high-density probe SNP-array approach to quantify the overall amount of chromosomal alterations in our tumor series. A total of 95 PTCs, including 34 DMs, 31 pN0s and 30 pN1s Control tumors, yielded a DNA amount and quality suitable for this analysis (Table 3). The overall average percentage of genome length affected by CNA in the whole series was 2.4 ± 4.5 %, with a range from 0 to 30.3 %.

**Table 3:** Genome-wide copy number variation analysis

	DMs	Controls	
Genome-wide CNA analysis^a^	(No. = 34) Mean ± SD (Range)	(No. = 61) Mean ± SD (Range)	*p* value
**Relative Length (as percentage of genome altered, %)**			
*Total CNA Events*	4.82 ± 6.53 (0.007, 30.3)	1.12 ± 1.99 (0.0 ,10.6)	0.002
*Duplications*	3.01 ± 4.63 (0, 22.9)	0.58 ± 1.30 (0 , 8.7)	0.005
*Amplifications*	0.04 ± 0.27 (0, 1.6)	0.001 ± 0.003 (0, 0.1)	0.32
*Deletions*	1.76 ± 2.40 (0, 9.1)	0.52 ± 1.36 (0, 8.3)	0.008
*Homozygous deletions*	0.002 ± 0.005 (0, 0.02)	0.001 ± 0.002 (0, 0.006)	0.52
**Number of Events (N)**			
*Total CNA Events*	31.9 ± 29.8 (2-138)	41.3 ± 80.8 (0, 547)	0.42
*Duplications*	23.5 ± 25.6 (1,118)	32.4 ± 67.2 (0, 444)	0.36
*Amplifications*	0.9 ± 3.9 (0, 23)	0.2 ± 0.5 (0, 2)	0.34
*Deletions*	7.1 ± 7.1 (1,33)	8.2 ± 7.1 (0, 101)	0.64
*Homozygous deletions*	0.5 ± 0.7 (0,2)	0.5 ± 0.6 (0,2)	0.52
**Genetic feature c**	**No. positive (%)**	**No. positive (%)**	
*Chr1q duplication*	12 (35.3)	2 (3.3)	5.1^-5^
*TERT duplication*	8 (23.5)	0 (0)	0.0001
*TERT mutation (C228T+C250T)*	13 (0.40.6)^*^	2 (3.4)	1.1^-5^
*THYT1 signature* *(Chr1q dup/TERT dup/TERT mutation)*	21 (63.6)	3 (5.1)	1.5^-9^

^a^Genome-wide CNA analysis was performed on 34 DMs and 61 controls. Within these samples, TERT promoter mutational status was correctly evaluated in 32 DMs and 59 controls, while 2 DMs and one control were not evaluable. One DM with unknown TERT status was Chr1 duplication-positive, therefore THYT1 signature status was correctly assigned in 33 DM PTCs and 59 Control PTCs.

**Figure 2:**
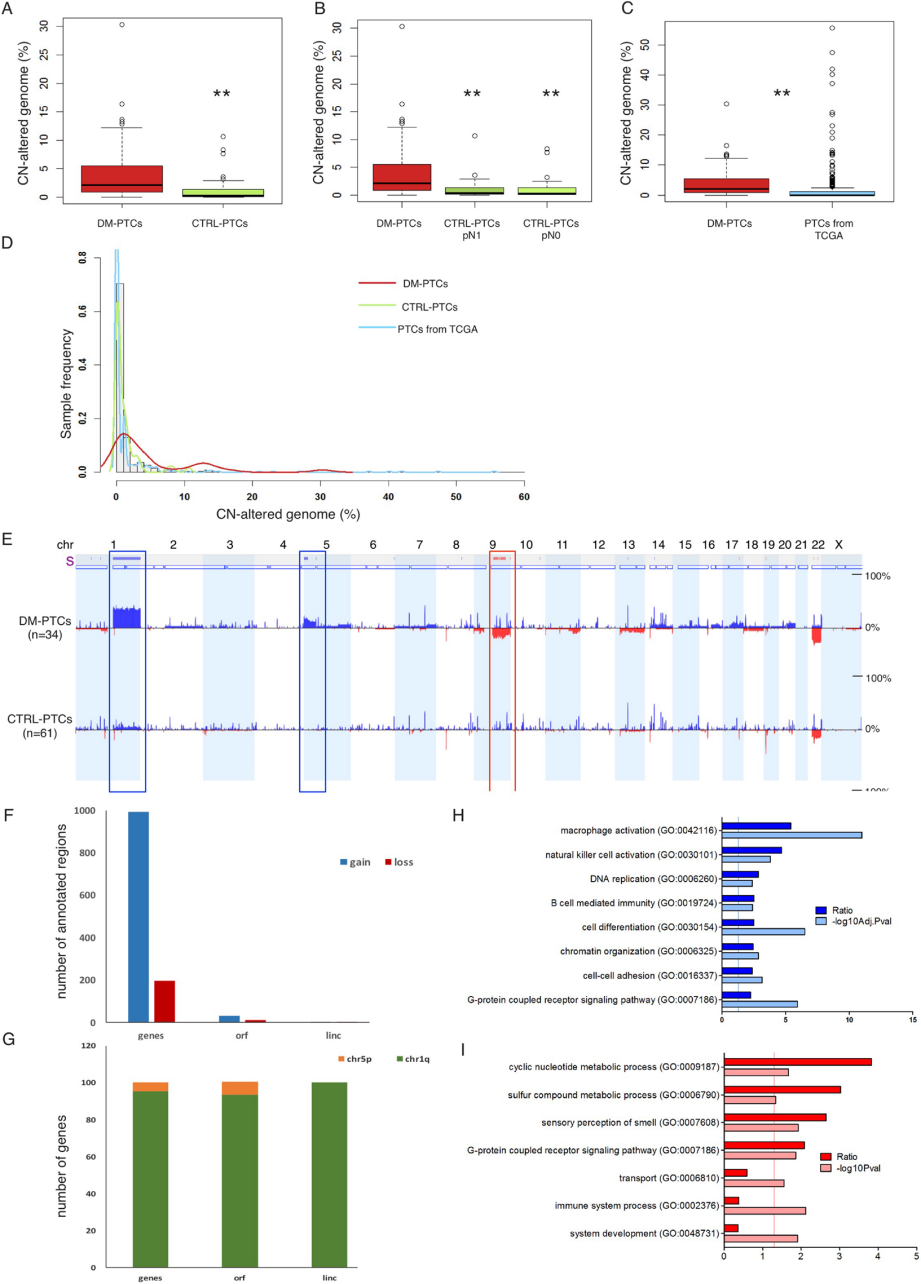
(**A**–**C**) Percentage of CN-altered genome in DM group vs. Control group (A); in DM group vs. the Control-pN1 and Control-pN0 groups (B); and in DM group from this study vs. 496 PTCs samples from The Cancer Genome Atlas data portal (C). (**D**) Distribution of the percentage of genome affected by CNA in DMs (red line), in Controls (green line), and in PTC samples from TCGA data portal (light blue line). (**E**) CNA frequency (from 0% to 50% as indicated) plotted by chromosome position, in the DM and in the Control groups; blue indicates duplications, dark blue indicates amplifications, red indicates deletions. The bar above the diagrams (labeled with “s”) indicates significant differences in CNA frequencies between two groups (Δ Freq.: 15%; *P* < 0.05). (**F**) Frequency of annotated regions within genomic fragments gained and lost in DMs as compared to Controls. (**G**) Distribution of annotated regions in Chr1q and Chr5p indicating that the vast majority of genes gained in DMs were located on Chr1q. (**H, I**) Gene ontology enrichment analysis of genes located in differential CNA regions of duplications (F) and of deletions (G) between DM and Control PTCs; dark bars indicate the R value (ratio between observed and expected gene frequency) and light bars indicate the negative log10 of the adjusted (F) or unadjusted (G) *P* values. Solid lines indicate the significance threshold of *P* = 0.05.

DMs showed a moderate extent of CNA (4.82 ± 4.5%, range 0.007–30.3%) that was significantly higher than in Controls (1.12 ± 1.99%, range 0.0–10.6%) (*P* = 0.002, Table 3, Figure 2A–2B). We also explored the distribution of CNA in pN0 and pN1 PTCs tumors in relation to DMs. We did not observe any difference in the overall CNA between the pN0 and pN1 groups; both showed a significant lower amount of genomic abnormalities as compared to DMs (Figure 2B).

We compared the distribution of the genomic CN damage using data available from the TCGA project: 496 PTCs 9 of which were DMs. For all these samples, data were retrieved and CNA profiles calculated. The average CN-altered genome relative length in the TCGA set was 1.54 ± 5.37 %, very similar to the value observed in our series of Controls (1.12 ± 1.99 %) and significantly lower than the average extent of CNA detected in our DMs (4.82 ± 6.53 %, *P* = 0.0007). Frequency distribution of the overall CNA revealed that the PTCs from TCGA and the Controls of our series had an overlapping profile that was profoundly different from the one of DMs (Figure 2C–2D). We also analyzed the genomic profile of seven distant metastases from well differentiated PTCs in lung (5), bronchus (1), and bone (1). Of these, two metastases were matched with a primary DM of our series. Overall distant metastases were characterized by a genomic CNA burden (4.8 ± 6.5 %) very similar to that of primary DM patients (Supplementary Figures 1–2).

Differential analysis of CNA profiles between DM and Control PTCs revealed that three genomic loci were preferentially gained or lost in DMs: duplication of Chr1q arm (*P* < 0.0001), duplication of a Chr5p harboring *TERT* locus (*P* = 0.0001), homozygous loss of Chr9q arm (*P* = 0.0004) (Figure 2E, Table 3). Loss of Chr9q was in strict association with Chr1q duplication (*P* = 2.2e-05), with only one sample showing Chr9q loss alone. Supplementary Tables 3 and 4 report the list of annotated regions within the regions significantly gained or lost in DMs as compared to Controls. A total of 994 proteins-coding genes, 31 open reading frames (ORFs) and 4 intergenic long non-coding RNAs (linc) were duplicated in DMs as compared to Controls while 217 proteins-coding genes 12 ORFs and 4 linc were lost in the aggressive tumors (Figure 2F). The majority of duplicated genes (about 95%) were located within Chr1q (Figure 2G). Gene Ontology (GO) analysis showed that gain regions were enriched for genes involved in immunity response, cell differentiation, chromatin organization and cell-cell adhesion (Figure 2H), while loss regions were enriched for genes involved in metabolic processes, cell signaling and immune process (Figure 2I) (Supplementary Table 3). Analysis of the genomic profile of the above mentioned seven metastases confirmed the presence of duplication of Chr1q, duplication of *TERT* locus and loss of Chr9q. In addition, metastasis acquired duplication of Chr17 (Supplementary Figures 1–2) in 4 out of 7 samples analyzed, suggesting that acquisition of this alteration is functional to the metastatic evolution of PTCs.

**Table 4:** Overall survival (OS) in DM patients^a^ by univariate and multivariate analysis

			Univariate analyses			Multivariate analyses^a^		
Covariates	No. (%)	deaths	HR	95% CI	*p* value	HR	95% CI	*p* value
CN-altered								
≤ 5.2 > 5.2	20 (61) 13 (49)	10 7	1 0.99	*Ref.* 0.46–2.15	0.985	1 2.29	- 0.64–8.16	0.201
THYT1								
Negative Positive	12 (36%) 21 (64%)	2 15	1 7.29	*Ref*. 1.63–32.57	0.001	1 6.81	- 1.31–35.36	0.022
Chr1_dup								
No Yes	21 (64%) 12 (36%)	7 10	1 5.30	*Ref.* 1.79–15.73	0.003	1 4.28	- 1.18–15.53	0.027
*TERT* duplication								
No Yes	25 (76%) 8 (24%)	12 5	1 2.82	*Ref.* 0.93–8.59	0.068	1 2.73	- 0.74–10.05	0.131
*TERT* mutation								
No Yes	19 (59%) 13 (41%)	7 9	1 2.02	*Ref.* 0.74–5.51	0.162	1 1.17	- 0.37–3.73	0.788

^a^ adjusted for Stage of disease (I–III; IV), histological variant (4 levels) and gender (Females, Males).

### The THYT1 genetic signature is a highly specific marker of distant metastasis in PTCs

**Figure 3:**
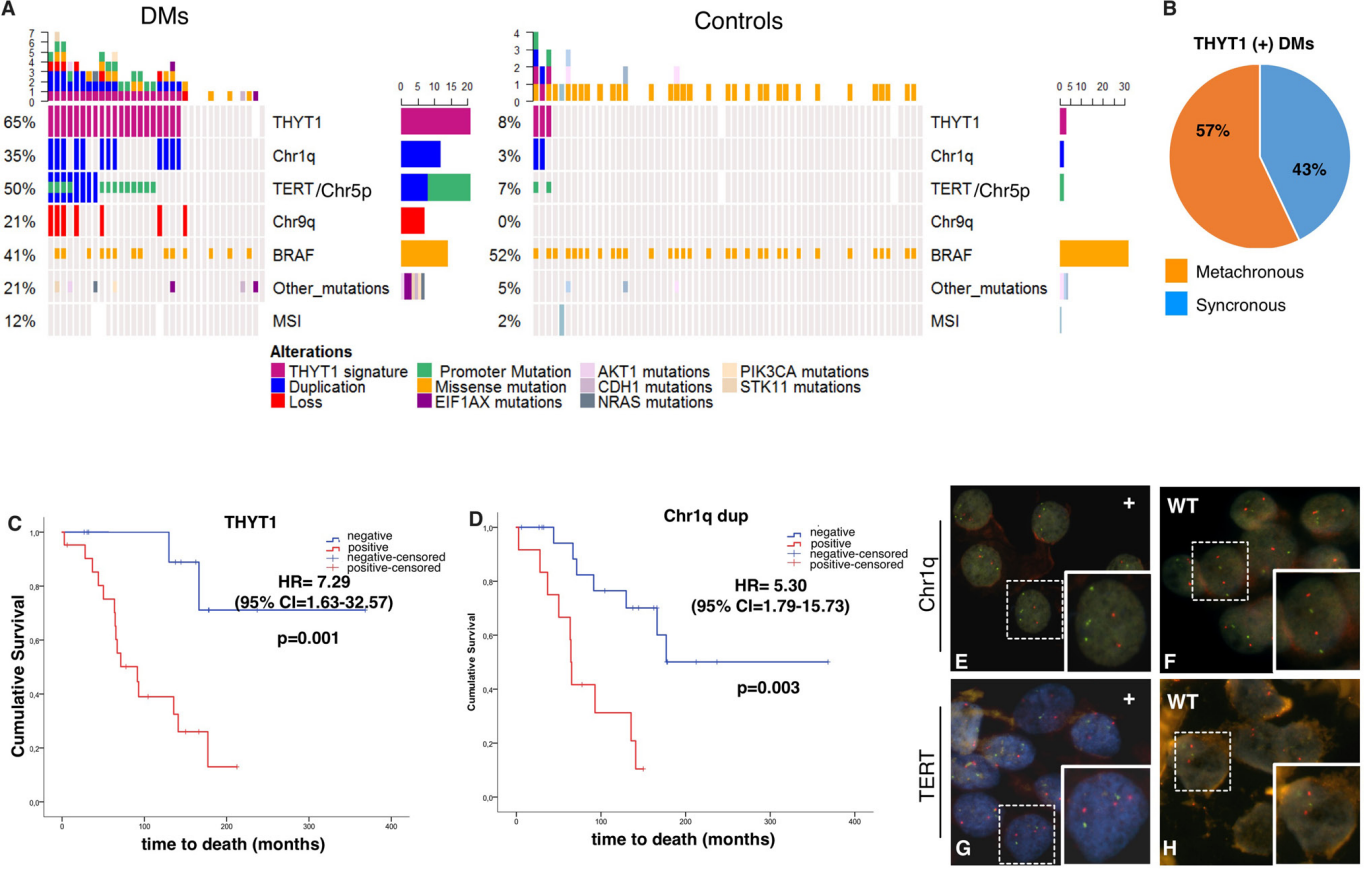
(**A**) Summary of the genetic and genomic alterations in the 95 PTCs analyzed with genome-wide SNP-array (34 DMs and 61 Controls). White rectangles indicate not available information. (**B**) Diagram of the distribution of synchronous (presenting metastasis at diagnosis) and metachronous (metastasis occurring at least one year from diagnosis) tumors within the THYT1 positive DMs set. C-D) Kaplan-Meier curves of thyroid carcinoma-specific survival by the presence of either the Chr1q duplication, *TERT* duplication or *TERT* mutation (THYT1 signature, (**C**) and Chr1q duplication status (**D**), in DMs (*n* = 48). Survival curves were compared by log-rank test, using the wild type (wt) as reference. *P* < 0.05 were considered as threshold for significance. (**E–H**) Fluorescent In Situ Hybridization to detect Chr1q duplication (E-F), and *TERT* locus duplication (G-H) in cytological specimens from FNA. In E-F green probe stains Chr1q, red probe stains Chr1p used as control. E is a representative DM sample positive for Chr1q duplication while F is a representative Control negative for Chr1q duplication. In G-H red probe stains TERT locus while green probe stains Chr5q used as control. G is a representative DM sample positive for TERT duplication while H is a representative DM sample negative for TERT duplication

Figure 3A summarizes the results of our analysis and shows the overall alterations found in DMs and in Controls subject to SNP-array analysis (34 DM PTCs and 61 Control PTCs). Duplication of Chr1q, duplication of Chr5p harboring the *TERT* locus and mutations in the *TERT* promoter showed the strongest association with the presence of distant metastasis. Combination of these genetic features defines a highly specific DM PTCs signature that we named Thyroid TERT Chr1q (THYT1). The occurrence of either one of these three alterations identified up to 65% of DM cases, while they were found in only 5% of Controls (*P* = 1.5^–9^) (Table 3) and defines positivity to THYT1. Co-existence of two or more of these three alterations was found in 26% of the DMs and only in one Control (1.6%). While the two Control PTCs that presented only one of the THYT1 alterations (namely mutations in the TERT promoter) did not show any evidence of disease at the last date of follow-up, the Control PTC with two alterations (namely mutations in the TERT promoter and duplication of Chr1q) presented local persistence of disease, confirming the high specificity of these alterations in highly aggressive well-differentiated PTCs. Noticeably, about 60% of THYT1 positive DMs developed metastasis more than one year after diagnosis and up to 71% of DMs with metachronous metastasis were positive for the THYT1 alterations (Figure 3B, Supplementary Tables 4–5), indicating that positivity for this signature may anticipate metastatic behavior even in the early phases of PTCs diagnosis.

### The THYT1 signature is an independent predictive marker of reduced survival probability in DM PTC patients

We assessed the correlation of THYT1 signature with patient survival probability. Since the THYT1 is strongly associated with the presence of distant metastasis and the presence of distant metastasis is a major determinant of patient mortality [4, 6, 7], we performed this analysis within the group of DM tumors. Noticeably, DM patients with tumors positive for the THYT1 signature displayed a significantly lower OS probability (HR = 7.29, *P* = 0.001) (Figure 3C–3D, Table 4) as compared with patients bearing THYT1 negative tumors. Presence of Chr1q duplication alone was as well significantly associated with reduced OS (HR = 5.30, *P* = 0.003). A lower OS was observed also for patients carrying *TERT* duplication (HR = 2.82, *P* = 0.068) although not statistically significant. *TERT* promoter mutations alone were not associated with OS in DM patients (HR = 2.02, *P* = 0.16, Table 4).

Because some clinical variables are known to be associated with patients’ outcome (Supplementary Table 6), we tested the independent effect of the molecular features in multivariate models correcting for gender, histological variant and pathologic stage at presentation. Multivariate analysis showed that the positivity to the THYT1 signature as well as Chr1q duplication were independently associated with OS (HR = 6.81, *p* = 0.022 and HR = 4.28, *p* = 0.027, for THYT1 and Chr1q, respectively, Table 4). The associations of the THYT1 signature with other clinical and pathological features are reported in Supplementary Table 7. THYT1 was significantly associated with age, higher stages and with lack of response to RAI. Overall these data demonstrated that the THYT1 signature is not only associated with the presence of distant metastasis but also correlates with a worse outcome of PTCs patients. A positive score for the THYT1 represents an independent risk factor for PTC patients, indicating the possibility of using the THYT1 signature as prognostic marker to predict aggressive clinical behavior of PTCs.

### THYT1 signature can be analyzed in pre-operative FNABs to predict aggressiveness in PTCs

Due to its high accuracy and documented safety, pre-operative fine needle aspiration biopsy (FNAB) has long been recognized as the gold standard technique in the diagnostic evaluation of thyroid nodules [13–18]. We assessed the possibility of applying the THYT1 signature in the pre-operative diagnosis of PTCs by Fluorescent in Situ Hybridization (FISH) (Figure 3E–3H). Fourteen and eight PTCs samples were positive at the SNP-array analysis for Chr1q and Chr5p (including the *TERT* locus) duplication respectively. FNAB cytological material was available for 10 out of 14 Chr1q duplication positive and 3 out of 8 *Chr5p* duplication positive tumors. As Control we selected 8 PTCs from our series without alteration in these loci as established by the SNP profile. Duplication of Chr1q and *TERT* was confirmed by FISH analysis in all positive samples, while none of the Controls displayed alterations and had a regular diploid status for these loci. This confirms the validity of our genome-wide analysis and indicates that the THYT1 signature can be easily and reliably evaluated in pre-surgical FNAB.

## DISCUSSION

Presence of distant metastasis characterizes less than 5% of well-differentiated PTCs but is the clinical feature majorly associated with a deadly outcome of the patients [4, 6, 7]. The rarity of widely metastatic PTCs and the slow course of this disease have so far made difficult the collection of sufficiently large cohorts of DM PTCs restraining the information about the genetic and molecular variables that underlie the metastatic progression of this tumor. In this study, we described for the first time the genomic landscape of a large series of DM PTCs. We showed that these tumors are characterized by a highly peculiar genetic asset that likely lies beneath their virulence and could represent a new prognostic tool in PTCs.

### Genetic aspects

High-density SNP profiles demonstrated that DMs are characterized by a moderate level of CNA and that a higher degree of genomic alteration predisposes to a reduced survival probability. Taking into account the overall length of altered genome, DMs seems to define a new subset of PTCs that stands in between the indolent differentiated PTCs and the highly aggressive, less differentiated forms of thyroid cancer that were reported to be characterized by a high degree of genomic abnormalities [9, 19]. Differential analysis between DMs and Controls identifies duplication of Chr1q, TERT locus in Chr5p and loss of Chr9q as distinctive features of DMs, suggesting a functional implication of these alterations in the mechanisms leading to metastatic spreading of PTCs. Amplification of Chr1q has been previously linked to aggressive features in thyroid cancer [9, 19, 20]. As well, mutations in the TERT promoter were described to occur with high frequency and to be associated with aggressiveness in different types of cancers including thyroid cancers [11, 21, 22]. In this work, we confirmed that TERT is a key gene in the metastatic progression of PTCs and we described for the first time that the amplification of the TERT genetic locus is strongly associated with the presence of distant metastasis and concur to reduced survival in PTC patients.

Seven distant metastases were also analyzed by SNP array profiling. Comparing the CNA profiles of DMs with the ones of distant metastases, we observed that the genomic assets of primary and metastatic lesions are very similar, both in terms of percentage of altered genome and types of alterations. The only exception was the amplification of Chr17, that was an exclusive feature of distant metastasis. This suggests that PTCs already acquire at the site of origin the chromosomal alterations that support distantly metastatic spreading. Tumor genetic heterogeneity in particular between primary and metastatic lesions is a hallmark of highly aggressive tumors and the consequence of DNA damage accumulation during uncontrolled proliferation. The genetic similarity observed between primary PTCs and metastasis seems to be in line with the relatively slow progression of PTCs and with the overall moderate degree of genomic instability that characterize these tumors. Noticeably, differences in the genetic assets of primary and corresponding metastasis is a major challenge for therapy choice in cancer patients. Our observation (even if limited to the analysis of chromosomal alterations) indicates that, in PTCs, discrepancy between the genetic asset of primary tumor and metastasis is not so marked as in other aggressive types of cancer.

### Clinical implications

While the incidence of thyroid cancer has grown steadily in recent decades in many countries, mortality rates remained essentially unchanged. This phenomenon is mainly determined by the improved performances of diagnostic technologies that facilitate the detection of early, small but likely indolent lesions. In the effort of developing large screening strategies, the inability to identify patients at high risk of true aggressive thyroid cancer disease increases the risk of “overdiagnosis” and “overtreatment”.

Therefore, development of new strategies to improve risk-based stratification of differentiated PTCs has become a major issue.

In this study, we described for the first time a signature of three genetic variables, that we named THYT1, that is strongly associated with the development of distant metastasis and with other major clinical features of aggressiveness in PTCs (like age, stage, and RAI refractoriness).

Noticeably, the THYT1 positivity not only identifies PTCs with synchronous metastasis but it is also a distinctive feature of tumors that developed metastasis several years later from the primary lesion, indicating that this signature may be a useful tool to foresee the metastatic progression of PTCs even in the early phases of tumor diagnosis.

Furthermore, we observed that the THYT1 signature is strongly associated with reduced overall survival. THYT1 positive DM patients had 7-fold higher risk to die as compared with THYT1 negative patients, a finding that was confirmed in multivariate analysis. THYT1 signature stood as independent predictor of negative outcome even when adjusted for strong prognostic feature such disease stage. To the best of our knowledge the THYT1 is the first genetic signature holding such prognostic value in well-differentiated PTCs.

Validation studies of our observations in larger and independent series of well-differentiated PTCs are of course needed. Nevertheless, our data lay the basis for the possible application of the THYT1 signature in PTC prognosis and potentially decision making.

To counteract the largely diffuse problem of PTCs overtreatment, a more conservative surgical management and a selective use of RAI has recently been encouraged [23]. Nevertheless, the choice of the appropriate therapeutic and follow-up strategy may be challenging, especially in those patients currently classified as at an intermediate prognostic risk, according to ATA guidelines [24]. Our analysis indicates that the THYT1 signature could be a very useful tool for identifying those patients likely to progress towards an aggressive and metastatic disease. The application of the THYT1 signature in the diagnostic setting of PTCs could improve the risk-based stratification of patients, and possibly allowing patient-tailored management strategies. In particular, this signature could accurately select high risk patients for whom a more radical surgical approach and a more intensive follow-up schedule should be warrant. As well, future trials assessing less aggressive therapeutic approaches on PTC patients [25–27] could benefit from the exclusion of patients with an unfavorable genetic profile, likely to jeopardize the results of long term prospective studies.

Molecular tests are currently used in the pre-surgical diagnostic setting to assist pathologists in discriminating between benign or malignant thyroid lesions [15–18, 28]. Here we showed that the THYT1 positivity can be reliably scored using routine techniques on FNAB materials, proving the feasibility of using this test also in the pre-surgical setting.

We are aware that the conclusion of our study needs to be corroborated by robust and consistent evidence from an independent series of patients that will allow validation of this signature. Nevertheless, the strength of our data suggests that the THYT1 signature is worthy to be considered as a potential negative prognostic marker in patients with newly diagnosed PTC.

## MATERIALS AND METHODS

### Patients and study design

The thyroid tumor archive of the Arcispedale S. Maria Nuova - IRCCS (ASMN-IRCCS), Reggio Emilia, Italy, includes 2,937 primary thyroid incident cancers cases diagnosed between 1978 and 2015. They are staged according to the AJCC Cancer Staging Manual, 7th edition. 2,594 are classified as differentiated papillary thyroid carcinoma, 210 as follicular and Hurthle cell carcinomas, 45 as poorly differentiated carcinomas, 38 as anaplastic carcinomas, and 95 as medullary carcinomas. PTCs with a diameter < 1cm at diagnosis (*n* = 1,123), with a follow up < 7 years (*n* = 739) and patients lost to follow up (*n* = 199) were excluded, leaving 488 PTCs available as a base for Controls selection. During the study period, 50 cases developed distant metastasis as documented either by clinical or histopathological records. The 438 eligible PTC Controls were classified according to their pN status. 191 PTCs were pNx and were excluded (Figure 1). Of the remaining, 86 were pN0 while 161 were pN1. Fifty pN0 patients and 48 pN1 patients (27 pN1a and 21 pN1b) were selected and matched by age at diagnosis (± 5 years) with DM cases.

Formalin fixed embedded (FFPE) tumor specimens stored in the pathology unit repository were retrieved for analysis. The flowchart of patient selection is shown in Figure 1. The research was conducted upon the approval from the Ethical Committee of the ASMN-IRCCS.

### DNA extraction and targeted next-generation sequencing analysis

For all selected case and Control patients, formalin fixed paraffin embedded (FFPE) specimens were retrieved from the pathology unit archive. An Hematoxilin and Eosin (H&E) staining was performed for each sample to confirm the morphology and to guide sample microdissection for genetic analysis. For each sample, ten 5μm thick slices were dissected under microscopy guidance to ensure a tumor cells content above 80% and used for DNA extraction. For each patient, a FFPE specimen from normal (non-tumoral) thyroid tissue was also retrieved and used for DNA extraction. Moreover, DNA was extracted from FFPE tissue of seven distant metastases, of which two were derived from primary DMs retrieved for this study, and 5 were derived from primary PTCs not available in our archive.

DNA was extracted using the FFPE Plus LEV DNA Purification Kit (Promega) and the Maxwell 16 instrument (Promega). DNA quality and quantity was assessed using Nanodrop and Qubit (Thermo Fisher Scientific, MA, USA).

Samples were subjected to the TruSight Tumor 26 genes panel (Illumina) resequencing. A total of 45 PTCs including 21 DM and 24 Control tumors yielded a DNA amount and quality suitable for this analysis. The panel consists in a 174-amplicon multiplexed targeted resequencing assay for the analysis of 26 genes. Targeted gene regions are shown in Supplementary Table 1. The generated libriaries were sequenced with the MiSeq Desktop Sequencer instrument (Illumina). Data were analyzed with the AmpliconDS protocol and with VariantStudio (Illumina) and IGV 2.3 (https://www.broadinstitute.org/igv/) softwares.

BRAF codon 600 and *TERT* promoter mutational status were investigated by direct sequencing as previously described [10–12] in 132 PTCs (DM = 48, Controls = 84) and 126 (DM = 44, Controls = 82) respectively. Finally, *EIF1AX* exon 1 and exon 2 were amplified with the following primers: Ex1_For:5′-CGCTACCCGGAAA GAAGTC-3′ and Ex1_Rev 5′-CTGGGTGACCTGCAAT CTAC-3′; Ex2_For:5′- TTAATGTCATTTACCTCCTTT TCTTT-3′ and Ex2_Rev:5′-AAAAATAAAGTCCCCA GCTAAAAA-3′ . Amplicons were sequenced using 454 GS-Junior Next Generation Sequencer (NGS – Roche, Mannheim, Germany) according to previously described protocol. A total of 141 PTCs were successfully sequenced for *EIF1AX* ( DM = 49, Controls = 97). Only nucleotide variations observed in both strands, in at least 10 reads and in at least 5% of the total number of reads analyzed were considered for mutational call.

### Microsatellite instability analysis

To determine microsatellite instability (MSI) status of the tumors, indicative of the impairment of the mismatch repair system in cancer, we investigated 13 polymorphic markers (BAT26, D17S250, TGFBR2, D2S123, D5S346, MybT22, BAT25, D18S58, MT1XT20, BAT40, NR21, NR24, CAT25) in neoplastic and corresponding non-neoplastic tissues. Moreover, three tetranucleotide markers (CSF1PO, D7S820, D18S51) were analyzed to confirm that tumor and non-neoplastic specimens derived from the same patient. MSI analysis was performed using with CEQ2000XL Genetic Analyzer (Beckman Coulter), and results analyzed using the CEQ fragment analysis software (Beckman Coulter). The presence of fluorescent peaks differently sized between tumoral and non-neoplastic profiles was interpreted as MSI. Tumors were classified as: i) MSS (microsatellite stable), when absence of instability for any tested locus was observed. ii) MSI-L (low MSI), when less than 30% but at least one MSI marker were unstable; iii) MSI-H (high MSI), if instable markers were greater than or equal to 30%. MSI was successfully quantified in 132 PTCs (DM = 40, Controls = 92).

### Copy number alterations (CNA)

The genome-wide analysis of CNA was performed by Cogentech Scarl (Milano, Italy) using the SNP array Oncoscan FFPE Kit (Affymetrix, Santa Clara, CA), as previously described [29]. Data are available at www.ebi.ac.uk/arrayexpress (Accession number:E-MTAB-5194).

Oncoscan data were processed using Nexus Express for Oncoscan 3 (Biodiscovery, El Segunda, CA), with the SNP-FASST2 segmentation algorithm, based on a hidden Markov model, and quadratic weave correction. Threshold for significance was set to 1 × 10^–7^ and a minimum of 10 probes were required to create a segment. Intensities > 0.1 and < –0.15 were set as duplications and hemizygous deletions, while intensities > 0.7 and < –1.1 were set as high gains (amplification) and homozygous deletions, respectively.

Allele ratio threshold was set to 0.4 for heterozygous imbalance, and a minimum of 85 % probes with allelic ratio above 0.8 in a segment of at least 500 Kb was required for homozygosity calling (LOH).

The chromosome CNA profiles were compared between PTCs groups using Nexus Express (“comparison” process), setting a adjusted *P*-value threshold of 0.05. Frequency threshold for calling was 25% in the overall sample, and minimum difference in frequency was set to 15%.

Protein-coding genes located in the differential minimal common regions of CNA were extracted with Galaxy (https://usegalaxy.org/) and the gene ontology enrichment analysis was performed by the online tool Panther ver11 (http://pantherdb.org/).

### TCGA data analysis

Level 3 segmented copy number data files from SNP-array experiments for 496 PTC and relative clinical data were retrieved by The Cancer Genome Atlas (TCGA) Data Portal website (http://cancergenome.nih.gov/). According to the clinical information available, 272 PTCs were pM0, 9 PTCs were pM1 and 201 were pMx (14 sample had unavailable pM status). For each sample, somatic CNV segments defined by at least 5 probes were selected. Average intensities > 0.1, and < –0.15 were set as duplications and deletions, respectively and the relative length of CN-altered genome was calculated for each sample.

### Statistical analysis

Descriptive statistics were computed for DM and Control patients and reported as means (± standard deviation, SD) for continuous variables. For categorical variables absolute and relative frequencies were reported. Clinico-pathological parameters measured in DM and Control patients were compared by using the Student's t test for continuous variables and the Chi^2^ or Fisher's exact test for categorical variables.

The Kaplan-Meier survival function was used to estimate overall survival (OS) and generate survival curves. To this purpose, time to death was defined as the difference between the date of death or end of follow up (for patients alive at the end of follow up) and the date of first diagnosis of thyroid cancer and expressed in months. The Cox proportional hazards model was used to compute the hazard ratio point estimates (HR) and their 95%CI. The log-rank test was used to assess the difference between OS in both univariate and multivariate analyses. In multivariate Cox's regression HR estimates were adjusted for stage of disease, histological variant, and gender. The threshold for statistical significance was set at *P* < 0.05. Statistical analysis was performed using IBM-SPSS version 23.

### Fluorescent *in situ* hybridization in FNA samples

Fluorescent In situ Hybridization (FISH) with probes located on Chr1q and on *TERT* locus was exploited to detect the presence of duplications in PTCs samples. FISH analysis was performed on cytological smears from Fine Needle Aspiration Biopsy (FNAB), stained with May-Gruenwald Giemsa as previously described [30]. The following probes were used: KBI-10709 TERT (5p15)/5q31 (Kreatech Diagnostics, US) for the analysis of *TERT* locus duplication; Vysis 1p36 / 1q25 probes (Abbott Molecular, Illinois, US) for the analysis of Chr1q duplication

## SUPPLEMENTARY MATERIALS FIGURES AND TABLES




