# Supplementary material for: Genome-wide profiling identifies the THYT1 signature as a distinctive feature of widely metastatic Papillary Thyroid Carcinomas
Source: Oncotarget. 2017 Dec 1;9(2):1813–25. doi: 10.18632/oncotarget.22805 (PMC5788601; doi:10.18632/oncotarget.22805)
Supplement: Supplementary file 1 [file oncotarget-09-1813-s001.pdf]

Genome-wide profiling identifies the THYT1 signature as a distinctive feature of widely metastatic Papillary Thyroid Carcinomas

SUPPLEMENTARY MATERIALS

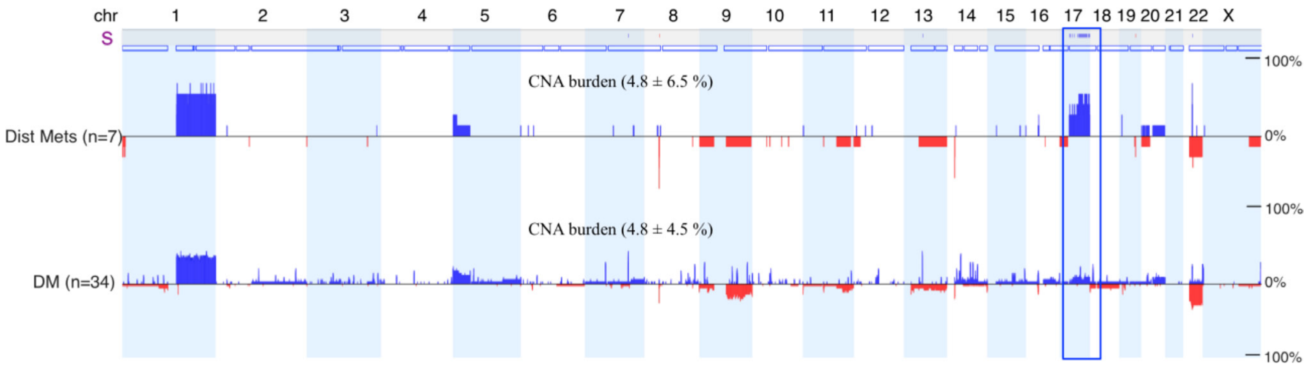

Supplementary Figure 1: Frequency plot of CNA by chromosome position in DMs (primary tumors) vs. distant metastases; the bar above the diagrams (labeled with "s") indicates significant differences in CNA frequencies between two groups (Δ Freq.: 15%; $P < 0.05$).

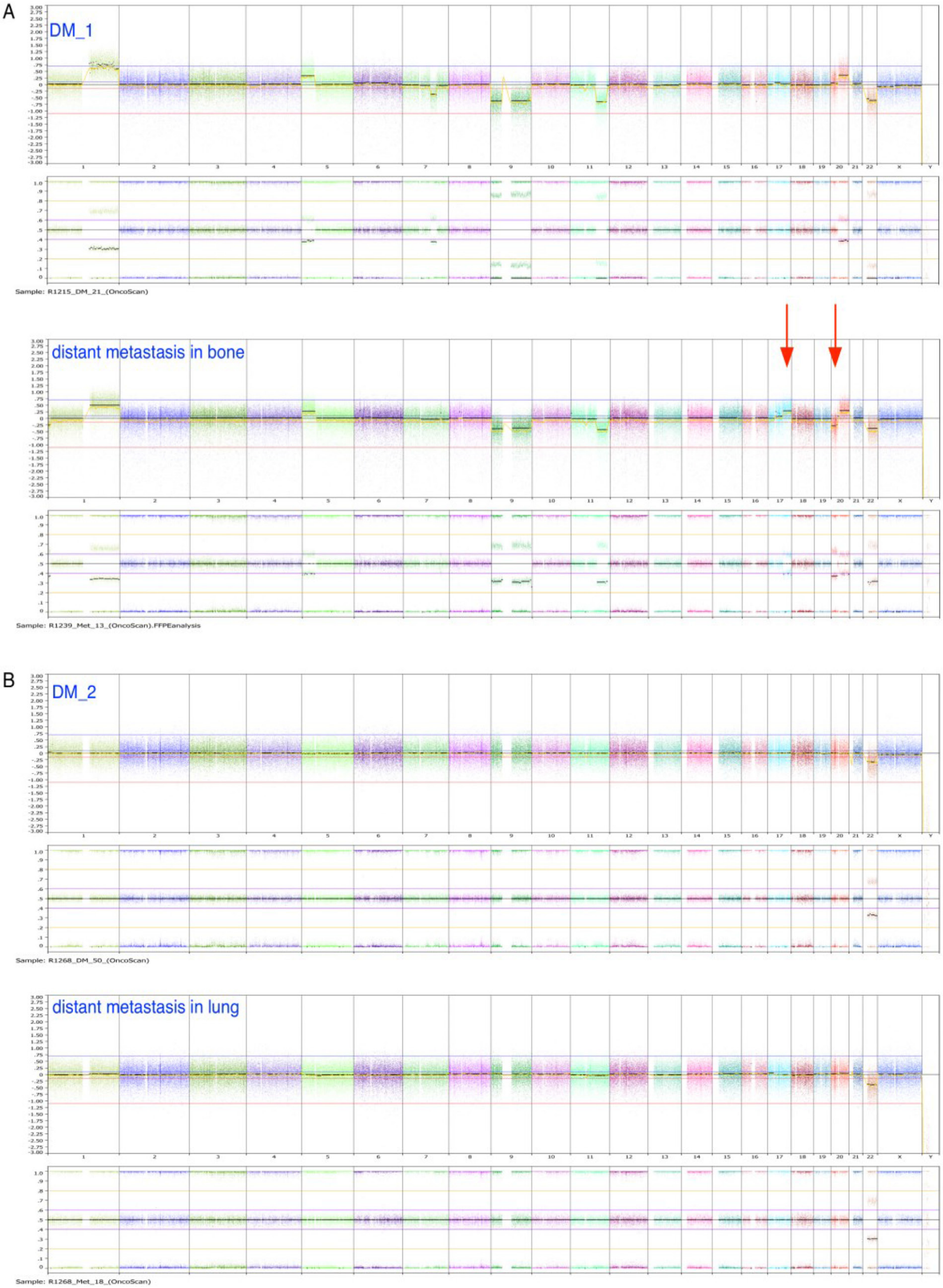

Supplementary Figure 2: (**A**) Genome-wide alteration profiles of a primary tumor from a 63 years old woman and a corresponding distant metastasis developed in bone after three years showed that metastasis profile resembled the primary lesion, but showed specific gain of chr 17 and loss of chr 21p arm; (**B**) Genome-wide alteration profiles of a primary PTCs from a 36-years old woman and the corresponding distant metastases developed in lung after 30 years from diagnosis: in this case, the only alteration in the primary was the loss of Chr22, which was found also in the metastasis. The upper plots show gains or losses of chromosomal material, the lower plots show the loss of heterozygosity, red arrows indicate alterations acquired at the metastatic site.

Supplementary Table 1: List of the 26 genes covered by the TruSight Tumor Panel (Illumina) used in the study

Genes	Exons
AKT1	3
ALK	23
APC	17
BRAF	11,15
CDH1	8,9,12
CTNNB1	3
EGFR	18, 19, 20, 21
ERBB2	23
FBXW7	7, 8 ,9 , 10 ,11 (c.)
FGFR2	7
FOXL2	1 (p.)
GNAQ	4, 5, 6
GNAS	6, 8
KIT	9, 11, 13, 17, 18
KRAS	2, 3, 4, 6
MAP2K1	2
MET	2 (p.), 5, 14, 16, 17, 18, 19, 21 (c.)
MSH6	5
NRAS	2, 3, 4, 5
PDGFRA	12, 14, 18
PIK3CA	2, 3, 8, 10, 21
PTEN	1, 2, 3, 4, 5
SMAD4	9, 12
SRC	14 (c.)
STK11	1 (c.), 4, 6, 8
TP53	2, 3, 4, 5, 6, 7, 8, 9, 10, 11

(p.); partial exon; (c.), coding region

Supplementary Table 2: Distribution of the detected mutations obtained by targeted NGS resequencing of 26 genes of the TruSight Tumor panel and EIF1AX, in DMs and Controls

	DMs	Controls	*P*
	**N(freq)**	**N (freq)**	
26 Genes TS panel	*n* = 21	*n* = 24	
AKT1 p.E49K	1 (.05)	0	
AKT1 p.E17K	1 (.05)	0	
APC p.L1129S	0	1 (.04)	
APC p.E1317Q	0	1 (.04)	
CDH1 p.A592T	1 (.05)	0	
KRAS p.A146T	0	1 (.04)	
MET p.E168D	0	1 (.04)	
NRAS p.Q61R	1 (.05)	0	
PIK3CA p.C420R	1 (.05)	0	
STK11 p.F354L	1 (.05)	0	
TP53 p.T253A	1 (.05)	0	
Total missense mutations	7	4	
Total mutated sample	7 (.33)	3 (.12)	0.15
EIF1AX exon 1-2	*n* = 49	*n* = 97	
p.M1I (c.3G>A)	0	1	
p.R13H (c.38 G>A)	0	1	
p.G15D (c.44G>A)	1	0	
p.E25K (c.73G>A)	0	1	
p.V27I (c.79G>A)	1	0	
p.K29E (c.85A>G)	0	1	
p.E30K(c.88G>A)	1	0	
p.D31G (c.92A>G)	0	1	
p.E34S (c.100G>A)	1	0	
Total missense mutations	4	5	
Total mutated sample	4 (.08)	4 (.04)	0.4

Supplementary Table 3: List of the genes located in differential CNA regions of duplications and of deletions between DMs and Controls and between DMs and distant metastases. See Supplementary_ Table_3

Supplementary Table 4: Genes in gain and loss regions

	location	Genes	ORF	LINC	LOC
	**total**	**994**	**31**	**4**	**0**
**GAIN**	**Chr1q**	**946**	**29**	**4**	**0**
	**Chr5p**	**48**	**2**	**0**	**0**
	**total**	**217**	**12**	**3**	**1**
**LOSS**	**Chr9p**	**197**	**11**	**3**	**1**
	**Chr22**	**20**	**1**	**0**	**0**

Supplementary Table 5: Frequency of principal genetic features in DM patients with metachronous or synchronous metastasis

**BRAF mutation**						
**mtastases_type**	**tot**	**mut**	**%**	**wt**	**%**	
metachronous	17	8	47,1	9	52,9	
synchronous	17	6	35,3	11	64,7	$P = 1$
**TERT mutation**						
**mtastases_type**	**tot**	**mut**	**%**	**wt**	**%**	
metachronous	17	8	47,1	9	52,9	
synchronous	17	5	29,4	12	70,6	$P = 0.296$
**TERT duplication**						
**mtastases_type**	**tot**	**Dup**	**%**	**wt**	**%**	
metachronous	17	5	29,4	12	70,6	
synchronous	17	3	17,6	14	82,4	$P = 0.688$
**Chr1q duplication**						
**mtastases_type**	**tot**	**Dup**	**%**	**wt**	**%**	
metachronous	17	7	41,2	10	58,8	
synchronous	17	5	29,4	12	70,6	$P = 0.721$
**ThyT1**						
**mtastases_type**	**tot**	**No**	**%**	**Yes**	**%**	
metachronous	17	5	29,4	12	70,6	
synchronous	17	7	41,2	9	52,9	$P = 0.481$

Supplementary Table 6: Survival analysis of clinical variable in DMs

Variables (No)	HR	95% CI	P[a]
Age	1.08	1.04–1.12	0.000
Gender			
Females (23)	1	-	
Males (17)	1.27	0.60–2.68	0.539
Histological Diagnosis[a]			
CPTCs (24)	1	-	
FV-PTC (8)	1.04	0.32–3.37	0.954
ST-PTC (2)	8.95	1.78–44.95	0.008
TCV-PTC (15)	4.19	1.71–1025	0.002
Pathologic Stage at Presentation			
I–III (18)	1		
IV (31)	2.09	0.90–4.92	0.088

Supplementary Table 7: Linear regression analysis of the percentage of genome affected by CNA and contingency analysis of THYT1 signature with clinical features of DMs

	Relative length of CN-altered genome			THYT1 signature		
**Variables**	**CE[a]**	**95% CI**	**P[b]**	**Negative**	**Positive**	**P[b]**
Age at diagnosis, y	0.15	0.04–0.26	**0.007**	36.4 ± 14.9	63.1 ± 12.7	**< 0.0001**
Sex			0.28			1
Females	reference			9 (.75)	15 (.71)	
Males	2.64	–2.34–7.63		3 (.25)	6 (.29)	
Histological Diagnosis[a]			**< 0.001**			0.13
CPTCs	reference			9 (.75)	7 (.33)	
TCV-PTCs	2.92	–0.83–6.68		2 (.17)	9 (.43)	
FV-PTCs	1.38	–3.56–6.32		1(.08)	4 (.19)	
ST-PTCs	27.47	17.42–37.43		0	1 (.05)	
Pathologic Stage at Presentation			0.13			**0.0007**
I	reference			2 (.16)	0	
II	-0.40	–10.49–9.69		6 (.50)	1 (.05)	
III	2.93	–7.29–13.17		0	7 (.33)	
IV	5.75	–3.78–15.29		4 (.33)	13 (.62)	
RAI refractoriness			0.06			**0.003**
n	reference			7 (.58)	1 (.06)	
y	5.13	–0.21–10.43		5(.41)	16 (.94)	
Extrathyroidal extension			0.257			1
n	reference			0	1 (.05)	
y	–7.60	–21.0–5.83		12 (1.0)	20 (.95)	
Vascular invasion			0.294			1
n	reference			3 (0.27)	4 (0.22)	
y	2.26	–2.07–6.60		8 (0.73)	14 (0.78)	

[a]Coefficient Estimate
[b]In bold significant *P*-value
