# Supplementary material for: Genome-wide profiling identifies the THYT1 signature as a distinctive feature of widely metastatic Papillary Thyroid Carcinomas
Source: Oncotarget. 2017 Dec 1;9(2):1813–25. doi: 10.18632/oncotarget.22805 (PMC5788601; doi:10.18632/oncotarget.22805)
Supplement: Supplementary file 2 [file oncotarget-09-1813-s002.docx]

**Supplementary Table S3**

| **GENES REGIONS IN GAIN REGIONS IN DM vs controls (n=995)** | | | |  | |  |  |  |  |  |  |
| --- | --- | --- | --- | --- | --- | --- | --- | --- | --- | --- | --- |
| Gene_symbol | Chr | Start |  | | Gene_symbol | Chr | Start |  | Gene_symbol | Chr | Start |
| ABCB10 | chr1 | 229652328 |  | | GPATCH2 | chr1 | 217600334 |  | PIGM | chr1 | 159997461 |
| ABL2 | chr1 | 179068461 |  | | GPATCH4 | chr1 | 156564099 |  | PIGR | chr1 | 207101866 |
| ACBD3 | chr1 | 226332379 |  | | GPR137B | chr1 | 236305831 |  | PIK3C2B | chr1 | 204391757 |
| ACBD6 | chr1 | 180257351 |  | | GPR161 | chr1 | 168048779 |  | PIP5K1A | chr1 | 151171020 |
| ACKR1 | chr1 | 159173802 |  | | GPR25 | chr1 | 200842082 |  | PKLR | chr1 | 155259083 |
| ACP6 | chr1 | 147119167 |  | | GPR37L1 | chr1 | 202092028 |  | PKP1 | chr1 | 201252579 |
| ACTA1 | chr1 | 229566992 |  | | GPR52 | chr1 | 174417211 |  | PLA2G4A | chr1 | 186798031 |
| ACTN2 | chr1 | 236849753 |  | | GPR89A | chr1 | 145764594 |  | PLD5 | chr1 | 242251688 |
| ADAM15 | chr1 | 155023747 |  | | GPR89B | chr1 | 147400505 |  | PLEKHA6 | chr1 | 204187978 |
| ADAMTS16 | chr5 | 5140442 |  | | GREM2 | chr1 | 240652872 |  | PLEKHG4B | chr5 | 140372 |
| ADAMTS4 | chr1 | 161159537 |  | | GUK1 | chr1 | 228327784 |  | PLEKHO1 | chr1 | 150122169 |
| ADAMTSL4 | chr1 | 150521844 |  | | H3F3A | chr1 | 226250407 |  | PLXNA2 | chr1 | 208195587 |
| ADAR | chr1 | 154554533 |  | | HAPLN2 | chr1 | 156589085 |  | PM20D1 | chr1 | 205797149 |
| ADCK3 | chr1 | 227127937 |  | | HAX1 | chr1 | 154245038 |  | PMF1 | chr1 | 156182778 |
| ADCY10 | chr1 | 167778624 |  | | HCN3 | chr1 | 155247217 |  | PMF1-BGLAP | chr1 | 156182778 |
| ADCY2 | chr5 | 7396342 |  | | HDGF | chr1 | 156711898 |  | PMVK | chr1 | 154897207 |
| ADIPOR1 | chr1 | 202909952 |  | | HEATR1 | chr1 | 236712304 |  | POGK | chr1 | 166808723 |
| ADORA1 | chr1 | 203096835 |  | | HFE2 | chr1 | 145413190 |  | POGZ | chr1 | 151375199 |
| ADSS | chr1 | 244571793 |  | | HHAT | chr1 | 210501595 |  | POLR3C | chr1 | 145592604 |
| AGT | chr1 | 230838271 |  | | HHIPL2 | chr1 | 222695601 |  | POLR3GL | chr1 | 145456235 |
| AHCTF1 | chr1 | 247002401 |  | | HIST2H2AA3 | chr1 | 149813784 |  | POU2F1 | chr1 | 167190065 |
| AHRR | chr5 | 304290 |  | | HIST2H2AB | chr1 | 149859018 |  | PPFIA4 | chr1 | 203020310 |
| AIDA | chr1 | 222841354 |  | | HIST2H2AC | chr1 | 149858524 |  | PPIAL4D | chr1 | 148201751 |
| AIM2 | chr1 | 159032274 |  | | HIST2H2BC | chr1 | 149821758 |  | PPIAL4E | chr1 | 148201751 |
| AKT3 | chr1 | 243651534 |  | | HIST2H2BE | chr1 | 149856009 |  | PPOX | chr1 | 161136180 |
| ALDH9A1 | chr1 | 165631448 |  | | HIST2H2BF | chr1 | 149754244 |  | PPP1R12B | chr1 | 202317829 |
| ANGEL2 | chr1 | 213165523 |  | | HIST2H3A | chr1 | 149824180 |  | PPP1R15B | chr1 | 204372491 |
| ANGPTL1 | chr1 | 178818669 |  | | HIST2H4A | chr1 | 149804220 |  | PPP2R5A | chr1 | 212458878 |
| ANKH | chr5 | 14704908 |  | | HIST3H2A | chr1 | 228645064 |  | PRCC | chr1 | 156737273 |
| ANKRD33B | chr5 | 10564434 |  | | HIST3H2BB | chr1 | 228645807 |  | PRDX6 | chr1 | 173446485 |
| ANKRD34A | chr1 | 145470507 |  | | HIST3H3 | chr1 | 228612545 |  | PRELP | chr1 | 203444882 |
| ANKRD35 | chr1 | 145549208 |  | | HLX | chr1 | 221052742 |  | PRG4 | chr1 | 186265404 |
| ANKRD36BP1 | chr1 | 168214818 |  | | HMCN1 | chr1 | 185703682 |  | PRKAB2 | chr1 | 146626684 |
| ANKRD45 | chr1 | 173577474 |  | | HNRNPU | chr1 | 245013601 |  | PROX1 | chr1 | 214161277 |
| ANP32E | chr1 | 150190716 |  | | HORMAD1 | chr1 | 150670534 |  | PRPF3 | chr1 | 150293927 |
| ANXA9 | chr1 | 150954498 |  | | HRNR | chr1 | 152184551 |  | PRR9 | chr1 | 153190059 |
| APCS | chr1 | 159557615 |  | | HSD11B1 | chr1 | 209859524 |  | PRRC2C | chr1 | 171454665 |
| APH1A | chr1 | 150237798 |  | | HSD17B7 | chr1 | 162760495 |  | PRRX1 | chr1 | 170633312 |
| APOA1BP | chr1 | 156561557 |  | | HSPA6 | chr1 | 161494329 |  | PRSS38 | chr1 | 228003417 |
| APOA2 | chr1 | 161192082 |  | | HSPA7 | chr1 | 161575848 |  | PRUNE | chr1 | 150980972 |
| APOBEC4 | chr1 | 183615410 |  | | IARS2 | chr1 | 220267454 |  | PSEN2 | chr1 | 227058272 |
| AQP10 | chr1 | 154293591 |  | | IBA57 | chr1 | 228353428 |  | PSMB4 | chr1 | 151372040 |
| ARF1 | chr1 | 228270360 |  | | IBA57-AS1 | chr1 | 228351786 |  | PSMD4 | chr1 | 151227196 |
| ARHGAP30 | chr1 | 161016731 |  | | ICE1 | chr5 | 5422785 |  | PTGS2 | chr1 | 186640943 |
| ARHGEF11 | chr1 | 156904631 |  | | IER5 | chr1 | 181057637 |  | PTPN14 | chr1 | 214522038 |
| ARHGEF2 | chr1 | 155916629 |  | | IFI16 | chr1 | 158979681 |  | PTPN7 | chr1 | 202116140 |
| ARID4B | chr1 | 235330209 |  | | IGFN1 | chr1 | 201159952 |  | PTPRC | chr1 | 198608097 |
| ARL8A | chr1 | 202102531 |  | | IGSF8 | chr1 | 160061128 |  | PVRL4 | chr1 | 161040780 |
| ARNT | chr1 | 150782180 |  | | IGSF9 | chr1 | 159896828 |  | PYCR2 | chr1 | 226107576 |
| ARPC5 | chr1 | 183595327 |  | | IKBKE | chr1 | 206643585 |  | PYGO2 | chr1 | 154929501 |
| ARV1 | chr1 | 231114822 |  | | IL10 | chr1 | 206940947 |  | PYHIN1 | chr1 | 158901336 |
| ASCL5 | chr1 | 201083080 |  | | IL19 | chr1 | 206972214 |  | QSOX1 | chr1 | 180123967 |
| ASH1L | chr1 | 155305051 |  | | IL20 | chr1 | 207039153 |  | RAB13 | chr1 | 153954092 |
| ASPM | chr1 | 197053256 |  | | IL24 | chr1 | 207070787 |  | RAB25 | chr1 | 156030965 |
| ASTN1 | chr1 | 176826440 |  | | IL6R | chr1 | 154377668 |  | RAB29 | chr1 | 205737113 |
| ATF3 | chr1 | 212738675 |  | | ILDR2 | chr1 | 166882440 |  | RAB3GAP2 | chr1 | 220321609 |
| ATF6 | chr1 | 161736033 |  | | ILF2 | chr1 | 153634263 |  | RAB4A | chr1 | 229406808 |
| ATP1A2 | chr1 | 160085519 |  | | INSRR | chr1 | 156810664 |  | RABGAP1L | chr1 | 174128551 |
| ATP1A4 | chr1 | 160121351 |  | | INTS3 | chr1 | 153700566 |  | RABIF | chr1 | 202847409 |
| ATP1B1 | chr1 | 169075946 |  | | INTS7 | chr1 | 212113740 |  | RALGPS2 | chr1 | 178694281 |
| ATP2B4 | chr1 | 203595914 |  | | IPO9 | chr1 | 201798287 |  | RASAL2 | chr1 | 178062863 |
| ATP6V1G3 | chr1 | 198492351 |  | | IQGAP3 | chr1 | 156495196 |  | RASSF5 | chr1 | 206680862 |
| ATP8B2 | chr1 | 154298035 |  | | IRF2BP2 | chr1 | 234740014 |  | RBBP5 | chr1 | 205055269 |
| AVPR1B | chr1 | 206224282 |  | | IRF6 | chr1 | 209958967 |  | RBM34 | chr1 | 235294497 |
| AXDND1 | chr1 | 179334854 |  | | IRX1 | chr5 | 3596167 |  | RBM8A | chr1 | 145507556 |
| B3GALNT2 | chr1 | 235610504 |  | | IRX2 | chr5 | 2746278 |  | RC3H1 | chr1 | 173900221 |
| B3GALT2 | chr1 | 193147859 |  | | ISG20L2 | chr1 | 156692412 |  | RCOR3 | chr1 | 211432707 |
| B4GALT3 | chr1 | 161141099 |  | | ITGA10 | chr1 | 145524989 |  | RCSD1 | chr1 | 167599473 |
| BATF3 | chr1 | 212859758 |  | | ITLN1 | chr1 | 160846329 |  | RD3 | chr1 | 211649863 |
| BCAN | chr1 | 156611739 |  | | ITLN2 | chr1 | 160914815 |  | REN | chr1 | 204123943 |
| BCL9 | chr1 | 147013270 |  | | ITPKB | chr1 | 226819390 |  | RFWD2 | chr1 | 175913961 |
| BECN1P1 | chr1 | 242121068 |  | | IVL | chr1 | 152881038 |  | RFX5 | chr1 | 151313115 |
| BGLAP | chr1 | 156211950 |  | | IVNS1ABP | chr1 | 185265521 |  | RGL1 | chr1 | 183605181 |
| BLZF1 | chr1 | 169337193 |  | | JMJD4 | chr1 | 227918889 |  | RGS1 | chr1 | 192544856 |
| BNIPL | chr1 | 151009028 |  | | JTB | chr1 | 153946744 |  | RGS13 | chr1 | 192605267 |
| BOLA1 | chr1 | 149871154 |  | | KCNH1 | chr1 | 210851656 |  | RGS16 | chr1 | 182567757 |
| BPNT1 | chr1 | 220230823 |  | | KCNJ10 | chr1 | 160007256 |  | RGS18 | chr1 | 192127591 |
| BRD9 | chr5 | 863849 |  | | KCNJ9 | chr1 | 160051359 |  | RGS2 | chr1 | 192778168 |
| BRINP2 | chr1 | 177140632 |  | | KCNK1 | chr1 | 233749749 |  | RGS21 | chr1 | 192286121 |
| BRINP3 | chr1 | 190066796 |  | | KCNK2 | chr1 | 215178884 |  | RGS4 | chr1 | 163038395 |
| BROX | chr1 | 222885894 |  | | KCNN3 | chr1 | 154669941 |  | RGS5 | chr1 | 163112088 |
| BTG2 | chr1 | 203274663 |  | | KCNT2 | chr1 | 196194909 |  | RGS7 | chr1 | 240938813 |
| BTNL10 | chr1 | 228698059 |  | | KCTD3 | chr1 | 215740734 |  | RGS8 | chr1 | 182615791 |
| C1orf100 | chr1 | 244515936 |  | | KDM5B | chr1 | 202696531 |  | RGSL1 | chr1 | 182419255 |
| C1orf101 | chr1 | 244624672 |  | | KIAA0040 | chr1 | 175126122 |  | RHBG | chr1 | 156338979 |
| C1orf105 | chr1 | 172389827 |  | | KIAA0907 | chr1 | 155882835 |  | RHOU | chr1 | 228780393 |
| C1orf106 | chr1 | 200860626 |  | | KIAA1614 | chr1 | 180882312 |  | RIIAD1 | chr1 | 151694012 |
| C1orf110 | chr1 | 162824086 |  | | KIAA1804 | chr1 | 233463513 |  | RIT1 | chr1 | 155867598 |
| C1orf111 | chr1 | 162343514 |  | | KIF14 | chr1 | 200520624 |  | RNASEL | chr1 | 182542768 |
| C1orf112 | chr1 | 169764549 |  | | KIF21B | chr1 | 200938513 |  | RNF115 | chr1 | 145610989 |
| C1orf115 | chr1 | 220863627 |  | | KIF26B | chr1 | 245318286 |  | RNF187 | chr1 | 228675067 |
| C1orf116 | chr1 | 207191865 |  | | KIFAP3 | chr1 | 169890469 |  | RNF2 | chr1 | 185014550 |
| C1orf131 | chr1 | 231359508 |  | | KIRREL | chr1 | 157963062 |  | RNPEP | chr1 | 201951765 |
| C1orf140 | chr1 | 221503269 |  | | KISS1 | chr1 | 204159468 |  | ROPN1L | chr5 | 10441973 |
| C1orf145 | chr1 | 228391206 |  | | KLHDC8A | chr1 | 205305192 |  | RORC | chr1 | 151778546 |
| C1orf186 | chr1 | 206238871 |  | | KLHDC9 | chr1 | 161068150 |  | RPRD2 | chr1 | 150336586 |
| C1orf189 | chr1 | 154171561 |  | | KLHL12 | chr1 | 202860229 |  | RPS27 | chr1 | 153963238 |
| C1orf198 | chr1 | 230972864 |  | | KLHL20 | chr1 | 173684079 |  | RPS6KC1 | chr1 | 213224574 |
| C1orf21 | chr1 | 184356149 |  | | KMO | chr1 | 241695433 |  | RPTN | chr1 | 152126070 |
| C1orf220 | chr1 | 178511930 |  | | KPRP | chr1 | 152730505 |  | RRNAD1 | chr1 | 156698262 |
| C1orf226 | chr1 | 162348695 |  | | KRTCAP2 | chr1 | 155141883 |  | RRP15 | chr1 | 218458628 |
| C1orf229 | chr1 | 247273461 |  | | LAD1 | chr1 | 201349965 |  | RUSC1 | chr1 | 155290639 |
| C1orf27 | chr1 | 186344889 |  | | LAMB3 | chr1 | 209788217 |  | RXFP4 | chr1 | 155911479 |
| C1orf35 | chr1 | 228288427 |  | | LAMC1 | chr1 | 182992594 |  | RXRG | chr1 | 165370158 |
| C1orf43 | chr1 | 154179176 |  | | LAMC2 | chr1 | 183155173 |  | RYR2 | chr1 | 237205701 |
| C1orf53 | chr1 | 197871681 |  | | LAMTOR2 | chr1 | 156024516 |  | S100A1 | chr1 | 153600872 |
| C1orf54 | chr1 | 150244686 |  | | LAX1 | chr1 | 203734283 |  | S100A10 | chr1 | 151955385 |
| C1orf56 | chr1 | 151020258 |  | | LBR | chr1 | 225589203 |  | S100A11 | chr1 | 152004981 |
| C1orf61 | chr1 | 156374054 |  | | LCE1A | chr1 | 152799948 |  | S100A12 | chr1 | 153346183 |
| C1orf68 | chr1 | 152691997 |  | | LCE1B | chr1 | 152784446 |  | S100A13 | chr1 | 153591275 |
| C1orf74 | chr1 | 209955661 |  | | LCE1C | chr1 | 152777310 |  | S100A14 | chr1 | 153586731 |
| C1orf95 | chr1 | 226736500 |  | | LCE1D | chr1 | 152769226 |  | S100A16 | chr1 | 153579366 |
| C2CD4D | chr1 | 151810338 |  | | LCE1E | chr1 | 152758752 |  | S100A2 | chr1 | 153533584 |
| C4BPA | chr1 | 207277606 |  | | LCE1F | chr1 | 152748847 |  | S100A3 | chr1 | 153519808 |
| C4BPB | chr1 | 207262211 |  | | LCE2A | chr1 | 152670839 |  | S100A4 | chr1 | 153516094 |
| C5orf38 | chr5 | 2752244 |  | | LCE2B | chr1 | 152658598 |  | S100A5 | chr1 | 153509622 |
| C5orf49 | chr5 | 7830490 |  | | LCE2C | chr1 | 152647790 |  | S100A6 | chr1 | 153507075 |
| CA14 | chr1 | 150230217 |  | | LCE2D | chr1 | 152635886 |  | S100A7 | chr1 | 153430219 |
| CACNA1E | chr1 | 181452685 |  | | LCE3A | chr1 | 152595309 |  | S100A7A | chr1 | 153388999 |
| CACNA1S | chr1 | 201008639 |  | | LCE3B | chr1 | 152586286 |  | S100A7L2 | chr1 | 153409470 |
| CACYBP | chr1 | 174968570 |  | | LCE3C | chr1 | 152573137 |  | S100A8 | chr1 | 153362507 |
| CADM3 | chr1 | 159141376 |  | | LCE3D | chr1 | 152551859 |  | S100A9 | chr1 | 153330329 |
| CAMK1G | chr1 | 209757044 |  | | LCE3E | chr1 | 152538174 |  | SCAMP3 | chr1 | 155225769 |
| CAMSAP2 | chr1 | 200708685 |  | | LCE4A | chr1 | 152681522 |  | SCCPDH | chr1 | 246887377 |
| CAPN2 | chr1 | 223889294 |  | | LCE5A | chr1 | 152483319 |  | SCNM1 | chr1 | 151138497 |
| CAPN8 | chr1 | 223714971 |  | | LCE6A | chr1 | 152815329 |  | SCYL3 | chr1 | 169822214 |
| CAPN9 | chr1 | 230883129 |  | | LEFTY1 | chr1 | 226073981 |  | SDC2 | chr8 | 97505881 |
| CASQ1 | chr1 | 160160284 |  | | LEFTY2 | chr1 | 226124297 |  | SDCCAG8 | chr1 | 243419306 |
| CCDC127 | chr5 | 204874 |  | | LELP1 | chr1 | 153175905 |  | SDE2 | chr1 | 226170402 |
| CCDC181 | chr1 | 169364107 |  | | LEMD1 | chr1 | 205350505 |  | SDHA | chr5 | 218337 |
| CCDC185 | chr1 | 223566714 |  | | LENEP | chr1 | 154966061 |  | SDHC | chr1 | 161284165 |
| CCSAP | chr1 | 229456751 |  | | LGALS8 | chr1 | 236681513 |  | SEC16B | chr1 | 177898241 |
| CCT3 | chr1 | 156278751 |  | | LGR6 | chr1 | 202163117 |  | SEC22B | chr1 | 145096406 |
| CCT5 | chr5 | 10250281 |  | | LHX4 | chr1 | 180199432 |  | SELE | chr1 | 169691780 |
| CD160 | chr1 | 145695797 |  | | LHX9 | chr1 | 197881634 |  | SELENBP1 | chr1 | 151336777 |
| CD1A | chr1 | 158223926 |  | | LIN9 | chr1 | 226418849 |  | SELL | chr1 | 169659805 |
| CD1B | chr1 | 158297739 |  | | LINC00303 | chr1 | 204001574 |  | SELP | chr1 | 169558087 |
| CD1C | chr1 | 158259562 |  | | LINC00467 | chr1 | 211556096 |  | SEMA4A | chr1 | 156119734 |
| CD1D | chr1 | 158149736 |  | | LINC00862 | chr1 | 200311671 |  | SEMA5A | chr5 | 9035137 |
| CD1E | chr1 | 158323485 |  | | LINC00869 | chr1 | 149576160 |  | SEMA6C | chr1 | 151104162 |
| CD244 | chr1 | 160799949 |  | | LINGO4 | chr1 | 151772764 |  | SERPINC1 | chr1 | 173872941 |
| CD247 | chr1 | 167399876 |  | | LIX1L | chr1 | 145477066 |  | SERTAD4 | chr1 | 210406194 |
| CD34 | chr1 | 208059882 |  | | LMNA | chr1 | 156052336 |  | SETDB1 | chr1 | 150898814 |
| CD46 | chr1 | 207925382 |  | | LMOD1 | chr1 | 201865583 |  | SF3B4 | chr1 | 149895208 |
| CD48 | chr1 | 160648535 |  | | LMX1A | chr1 | 165171103 |  | SFT2D2 | chr1 | 168195254 |
| CD55 | chr1 | 207494816 |  | | LOR | chr1 | 153232178 |  | SH2D1B | chr1 | 162365055 |
| CD5L | chr1 | 157800703 |  | | LPGAT1 | chr1 | 211916798 |  | SH2D2A | chr1 | 156776034 |
| CD84 | chr1 | 160510883 |  | | LRRC14B | chr5 | 191625 |  | SH3BP5L | chr1 | 249104650 |
| CDC42BPA | chr1 | 227177565 |  | | LRRC52 | chr1 | 165513477 |  | SHC1 | chr1 | 154934773 |
| CDC42SE1 | chr1 | 151023446 |  | | LRRC71 | chr1 | 156890423 |  | SHCBP1L | chr1 | 182868999 |
| CDC73 | chr1 | 193091087 |  | | LRRN2 | chr1 | 204586302 |  | SHE | chr1 | 154451953 |
| CDK18 | chr1 | 205473683 |  | | LY9 | chr1 | 160765863 |  | SHISA4 | chr1 | 201857796 |
| CELF3 | chr1 | 151672533 |  | | LYPD8 | chr1 | 248902716 |  | SIPA1L2 | chr1 | 232533711 |
| CENPF | chr1 | 214776531 |  | | LYPLAL1 | chr1 | 219347172 |  | SLAMF1 | chr1 | 160579608 |
| CENPL | chr1 | 173768687 |  | | LYSMD1 | chr1 | 151132223 |  | SLAMF6 | chr1 | 160454819 |
| CEP170 | chr1 | 243287729 |  | | LYST | chr1 | 235824330 |  | SLAMF7 | chr1 | 160708846 |
| CEP350 | chr1 | 179923907 |  | | MAEL | chr1 | 166944818 |  | SLAMF8 | chr1 | 159796478 |
| CEP72 | chr5 | 612404 |  | | MAP10 | chr1 | 232940637 |  | SLAMF9 | chr1 | 159921281 |
| CERS2 | chr1 | 150937648 |  | | MAP1LC3C | chr1 | 242158791 |  | SLC12A7 | chr5 | 1050488 |
| CFAP126 | chr1 | 161334520 |  | | MAPKAPK2 | chr1 | 206858364 |  | SLC19A2 | chr1 | 169433148 |
| CFAP45 | chr1 | 159842153 |  | | MARC1 | chr1 | 220960038 |  | SLC25A44 | chr1 | 156163722 |
| CFH | chr1 | 196621007 |  | | MARC2 | chr1 | 220921675 |  | SLC26A9 | chr1 | 205882176 |
| CFHR1 | chr1 | 196788860 |  | | MARCH2 | chr5 | 10353750 |  | SLC27A3 | chr1 | 153747767 |
| CFHR2 | chr1 | 196912933 |  | | MARK1 | chr1 | 220701524 |  | SLC30A1 | chr1 | 211748380 |
| CFHR3 | chr1 | 196743929 |  | | MCL1 | chr1 | 150547026 |  | SLC30A10 | chr1 | 220087605 |
| CFHR4 | chr1 | 196857143 |  | | MDM4 | chr1 | 204485506 |  | SLC35F3 | chr1 | 234040457 |
| CFHR5 | chr1 | 196946666 |  | | MED10 | chr5 | 6372038 |  | SLC39A1 | chr1 | 153931574 |
| CGN | chr1 | 151483861 |  | | MEF2D | chr1 | 156433512 |  | SLC41A1 | chr1 | 205758220 |
| CHD1L | chr1 | 146714290 |  | | METTL11B | chr1 | 170115187 |  | SLC45A3 | chr1 | 205626980 |
| CHI3L1 | chr1 | 203148058 |  | | METTL13 | chr1 | 171750760 |  | SLC50A1 | chr1 | 155107819 |
| CHIT1 | chr1 | 203185206 |  | | METTL18 | chr1 | 169761669 |  | SLC6A18 | chr5 | 1225469 |
| CHML | chr1 | 241792166 |  | | MEX3A | chr1 | 156041803 |  | SLC6A19 | chr5 | 1201709 |
| CHRM3 | chr1 | 239792372 |  | | MFSD4 | chr1 | 205538111 |  | SLC9A3 | chr5 | 473333 |
| CHRNB2 | chr1 | 154540256 |  | | MGST3 | chr1 | 165600109 |  | SLC9C2 | chr1 | 173469603 |
| CHTOP | chr1 | 153606457 |  | | MIA3 | chr1 | 222791443 |  | SMCP | chr1 | 152850797 |
| CIART | chr1 | 150254942 |  | | MIXL1 | chr1 | 226411318 |  | SMG5 | chr1 | 156219014 |
| CKS1B | chr1 | 154947117 |  | | MLLT11 | chr1 | 151032150 |  | SMG7 | chr1 | 183441505 |
| CLK2 | chr1 | 155232658 |  | | MNDA | chr1 | 158801167 |  | SMYD2 | chr1 | 214454564 |
| CMBL | chr5 | 10277706 |  | | MPC2 | chr1 | 167885912 |  | SMYD3 | chr1 | 245912641 |
| CNIH3 | chr1 | 224804178 |  | | MPZ | chr1 | 161274524 |  | SNAP47 | chr1 | 227922696 |
| CNIH4 | chr1 | 224544512 |  | | MPZL1 | chr1 | 167691186 |  | SNAPIN | chr1 | 153631129 |
| CNST | chr1 | 246729638 |  | | MR1 | chr1 | 181002560 |  | SNRPE | chr1 | 203830739 |
| CNTN2 | chr1 | 205012339 |  | | MROH9 | chr1 | 170904611 |  | SNX27 | chr1 | 151584661 |
| COA6 | chr1 | 234509182 |  | | MRPL24 | chr1 | 156707093 |  | SOAT1 | chr1 | 179262848 |
| COG2 | chr1 | 230778201 |  | | MRPL55 | chr1 | 228294379 |  | SOX13 | chr1 | 204042245 |
| COLGALT2 | chr1 | 183904965 |  | | MRPL9 | chr1 | 151732118 |  | SPATA17 | chr1 | 217804694 |
| COPA | chr1 | 160258376 |  | | MRPS14 | chr1 | 174982093 |  | SPATA45 | chr1 | 213003484 |
| COX20 | chr1 | 244998638 |  | | MRPS21 | chr1 | 150266261 |  | SPHAR | chr1 | 229440128 |
| CR1 | chr1 | 207669472 |  | | MSTO1 | chr1 | 155579960 |  | SPRR1A | chr1 | 152956563 |
| CR1L | chr1 | 207818457 |  | | MT1HL1 | chr1 | 237167402 |  | SPRR1B | chr1 | 153003678 |
| CR2 | chr1 | 207627644 |  | | MTMR11 | chr1 | 149900542 |  | SPRR2A | chr1 | 153028595 |
| CRABP2 | chr1 | 156669399 |  | | MTR | chr1 | 236958580 |  | SPRR2B | chr1 | 153042717 |
| CRB1 | chr1 | 197170591 |  | | MTRR | chr5 | 7869216 |  | SPRR2D | chr1 | 153012200 |
| CRCT1 | chr1 | 152486977 |  | | MTX1 | chr1 | 155178489 |  | SPRR2E | chr1 | 153065610 |
| CREB3L4 | chr1 | 153940314 |  | | MUC1 | chr1 | 155158299 |  | SPRR2F | chr1 | 153084612 |
| CREG1 | chr1 | 167510250 |  | | MYBPH | chr1 | 203136938 |  | SPRR2G | chr1 | 153122057 |
| CRNN | chr1 | 152381718 |  | | MYOC | chr1 | 171604556 |  | SPRR3 | chr1 | 152974222 |
| CRP | chr1 | 159682078 |  | | MYOG | chr1 | 203052256 |  | SPRR4 | chr1 | 152943127 |
| CRTC2 | chr1 | 153920147 |  | | NAV1 | chr1 | 201617449 |  | SPRTN | chr1 | 231473681 |
| CTNND2 | chr5 | 10971953 |  | | NBPF10 | chr1 | 145293370 |  | SPTA1 | chr1 | 158580495 |
| CTSE | chr1 | 206317458 |  | | NBPF11 | chr1 | 146032541 |  | SRD5A1 | chr5 | 6633499 |
| CTSK | chr1 | 150768683 |  | | NBPF12 | chr1 | 146373856 |  | SRGAP2 | chr1 | 206516199 |
| CTSS | chr1 | 150702671 |  | | NBPF14 | chr1 | 148250248 |  | SRGAP2B | chr1 | 206516199 |
| CYB5R1 | chr1 | 202931000 |  | | NBPF15 | chr1 | 148558187 |  | SRGAP2C | chr1 | 206516199 |
| DAP | chr5 | 10679341 |  | | NBPF20 | chr1 | 144146810 |  | SRP9 | chr1 | 225965514 |
| DAP3 | chr1 | 155658881 |  | | NBPF8 | chr1 | 144614958 |  | SSR2 | chr1 | 155978838 |
| DARS2 | chr1 | 173793796 |  | | NBPF9 | chr1 | 144614958 |  | STX6 | chr1 | 180941849 |
| DCAF6 | chr1 | 167905796 |  | | NCF2 | chr1 | 183524696 |  | SUCO | chr1 | 172501488 |
| DCAF8 | chr1 | 160185504 |  | | NCSTN | chr1 | 160313062 |  | SUSD4 | chr1 | 223394160 |
| DCST1 | chr1 | 155006281 |  | | NDUFS2 | chr1 | 161169104 |  | SV2A | chr1 | 149874871 |
| DCST2 | chr1 | 154991002 |  | | NEK2 | chr1 | 211831598 |  | SWT1 | chr1 | 185126191 |
| DDR2 | chr1 | 162602227 |  | | NEK7 | chr1 | 198126107 |  | SYT11 | chr1 | 155829259 |
| DDX59 | chr1 | 200613164 |  | | NENF | chr1 | 212606228 |  | SYT14 | chr1 | 210111518 |
| DEDD | chr1 | 161090768 |  | | NES | chr1 | 156638555 |  | SYT2 | chr1 | 202559724 |
| DEGS1 | chr1 | 224370909 |  | | NFASC | chr1 | 204797781 |  | TADA1 | chr1 | 166825748 |
| DENND1B | chr1 | 197473878 |  | | NHLH1 | chr1 | 160336860 |  | TAF1A | chr1 | 222731243 |
| DENND4B | chr1 | 153901976 |  | | NID1 | chr1 | 236139131 |  | TAF5L | chr1 | 229728865 |
| DESI2 | chr1 | 244816349 |  | | NIT1 | chr1 | 161087861 |  | TAGLN2 | chr1 | 159887896 |
| DHX9 | chr1 | 182808438 |  | | NKD2 | chr5 | 1009076 |  | TARBP1 | chr1 | 234527058 |
| DIEXF | chr1 | 210001311 |  | | NLRP3 | chr1 | 247579457 |  | TARS2 | chr1 | 150459839 |
| DISC1 | chr1 | 231762560 |  | | NME7 | chr1 | 169101767 |  | TAS2R1 | chr5 | 9629108 |
| DISP1 | chr1 | 222988430 |  | | NMNAT2 | chr1 | 183217371 |  | TATDN3 | chr1 | 212965169 |
| DNAH14 | chr1 | 225117355 |  | | NOS1AP | chr1 | 162039580 |  | TBCE | chr1 | 235530674 |
| DNAH5 | chr5 | 13690436 |  | | NOTCH2NL | chr1 | 145209112 |  | TBX19 | chr1 | 168250277 |
| DNM3 | chr1 | 171810617 |  | | NPHS2 | chr1 | 179519673 |  | TCHH | chr1 | 152078792 |
| DPM3 | chr1 | 155112366 |  | | NPL | chr1 | 182758583 |  | TCHHL1 | chr1 | 152056619 |
| DPT | chr1 | 168664694 |  | | NPR1 | chr1 | 153651163 |  | TDRD10 | chr1 | 154474694 |
| DSTYK | chr1 | 205111630 |  | | NR1I3 | chr1 | 161199455 |  | TDRD5 | chr1 | 179560747 |
| DTL | chr1 | 212208894 |  | | NR5A2 | chr1 | 199996729 |  | TDRKH | chr1 | 151744040 |
| DUSP10 | chr1 | 221874761 |  | | NSL1 | chr1 | 212899494 |  | TEDDM1 | chr1 | 182367251 |
| DUSP12 | chr1 | 161719557 |  | | NSUN2 | chr5 | 6599351 |  | TERT | chr5 | 1253286 |
| DUSP23 | chr1 | 159750758 |  | | NTPCR | chr1 | 233086369 |  | TEX35 | chr1 | 178482211 |
| DUSP27 | chr1 | 167064086 |  | | NTRK1 | chr1 | 156785541 |  | TFB2M | chr1 | 246703862 |
| DYRK3 | chr1 | 206808880 |  | | NUAK2 | chr1 | 205271190 |  | TGFB2 | chr1 | 218518675 |
| ECM1 | chr1 | 150480486 |  | | NUCKS1 | chr1 | 205681946 |  | THBS3 | chr1 | 155165378 |
| EDARADD | chr1 | 236557679 |  | | NUDT17 | chr1 | 145586492 |  | THEM4 | chr1 | 151843342 |
| EDEM3 | chr1 | 184659624 |  | | NUF2 | chr1 | 163291722 |  | THEM5 | chr1 | 151819576 |
| EFCAB2 | chr1 | 245133170 |  | | NUP133 | chr1 | 229577043 |  | TIMM17A | chr1 | 201924618 |
| EFNA1 | chr1 | 155100348 |  | | NUP210L | chr1 | 153965167 |  | TIPRL | chr1 | 168148082 |
| EFNA3 | chr1 | 155051347 |  | | NVL | chr1 | 224415035 |  | TLR5 | chr1 | 223282747 |
| EFNA4 | chr1 | 155036212 |  | | OAZ3 | chr1 | 151735444 |  | TMCC2 | chr1 | 205197037 |
| EGLN1 | chr1 | 231499496 |  | | OBSCN | chr1 | 228395830 |  | TMCO1 | chr1 | 165693527 |
| EIF2D | chr1 | 206764973 |  | | OCLM | chr1 | 186369703 |  | TMEM183A | chr1 | 202976533 |
| ELF3 | chr1 | 201979689 |  | | OLFML2B | chr1 | 161952981 |  | TMEM183B | chr1 | 202976535 |
| ELK4 | chr1 | 205577070 |  | | OPN3 | chr1 | 241756451 |  | TMEM206 | chr1 | 212537815 |
| ENAH | chr1 | 225674533 |  | | OPTC | chr1 | 203463270 |  | TMEM63A | chr1 | 226033232 |
| ENSA | chr1 | 150594598 |  | | OR10J1 | chr1 | 159409511 |  | TMEM79 | chr1 | 156252703 |
| EPHX1 | chr1 | 225997775 |  | | OR10J3 | chr1 | 159283459 |  | TMEM81 | chr1 | 205052256 |
| EPRS | chr1 | 220141941 |  | | OR10J5 | chr1 | 159504867 |  | TMEM9 | chr1 | 201103898 |
| ERO1LB | chr1 | 236378421 |  | | OR10K1 | chr1 | 158435351 |  | TMOD4 | chr1 | 151142462 |
| ESRRG | chr1 | 216676587 |  | | OR10K2 | chr1 | 158389717 |  | TNFAIP8L2 | chr1 | 151129104 |
| ETNK2 | chr1 | 204100188 |  | | OR10R2 | chr1 | 158449667 |  | TNFSF18 | chr1 | 173010359 |
| ETV3 | chr1 | 157094458 |  | | OR10T2 | chr1 | 158368311 |  | TNFSF4 | chr1 | 173152869 |
| ETV3L | chr1 | 157061834 |  | | OR10X1 | chr1 | 158548708 |  | TNN | chr1 | 175036993 |
| EXO1 | chr1 | 242011492 |  | | OR10Z1 | chr1 | 158576228 |  | TNNI1 | chr1 | 201372894 |
| EXOC3 | chr5 | 443333 |  | | OR11L1 | chr1 | 248004229 |  | TNNT2 | chr1 | 201328135 |
| EXOC8 | chr1 | 231468481 |  | | OR13G1 | chr1 | 247835419 |  | TNR | chr1 | 175291934 |
| F11R | chr1 | 160965000 |  | | OR14A16 | chr1 | 247978101 |  | TOMM20 | chr1 | 235272657 |
| F13B | chr1 | 197008320 |  | | OR14C36 | chr1 | 248512076 |  | TOMM40L | chr1 | 161195728 |
| F5 | chr1 | 169481191 |  | | OR14I1 | chr1 | 248844669 |  | TOR1AIP1 | chr1 | 179851176 |
| FAIM3 | chr1 | 207076630 |  | | OR1C1 | chr1 | 247920763 |  | TOR1AIP2 | chr1 | 179809101 |
| FAM105A | chr5 | 14581890 |  | | OR2AK2 | chr1 | 248128633 |  | TOR3A | chr1 | 179051111 |
| FAM129A | chr1 | 184760158 |  | | OR2B11 | chr1 | 247614330 |  | TP53BP2 | chr1 | 223967594 |
| FAM163A | chr1 | 179712297 |  | | OR2C3 | chr1 | 247693433 |  | TPM3 | chr1 | 154127779 |
| FAM173B | chr5 | 10225619 |  | | OR2G2 | chr1 | 247751661 |  | TPPP | chr5 | 659976 |
| FAM177B | chr1 | 222910557 |  | | OR2G3 | chr1 | 247768887 |  | TPR | chr1 | 186280785 |
| FAM189B | chr1 | 155216995 |  | | OR2G6 | chr1 | 248684947 |  | TRAF3IP3 | chr1 | 209929376 |
| FAM20B | chr1 | 178995073 |  | | OR2L13 | chr1 | 248100492 |  | TRAF5 | chr1 | 211499956 |
| FAM231D | chr1 | 149287450 |  | | OR2L2 | chr1 | 248201473 |  | TRIM11 | chr1 | 228581376 |
| FAM63A | chr1 | 150969300 |  | | OR2L3 | chr1 | 248223983 |  | TRIM17 | chr1 | 228595635 |
| FAM71A | chr1 | 212797788 |  | | OR2L5 | chr1 | 248185249 |  | TRIM46 | chr1 | 155146262 |
| FAM72A | chr1 | 206138910 |  | | OR2L8 | chr1 | 248112159 |  | TRIM58 | chr1 | 248020500 |
| FAM72C | chr1 | 206138439 |  | | OR2M2 | chr1 | 248343287 |  | TRIM67 | chr1 | 231298673 |
| FAM78B | chr1 | 166039255 |  | | OR2M3 | chr1 | 248366369 |  | TRIO | chr5 | 14143828 |
| FAM89A | chr1 | 231154703 |  | | OR2M4 | chr1 | 248402230 |  | TRIP13 | chr5 | 892968 |
| FASLG | chr1 | 172628147 |  | | OR2M5 | chr1 | 248308449 |  | TRMT1L | chr1 | 185087217 |
| FASTKD3 | chr5 | 7859271 |  | | OR2M7 | chr1 | 248486931 |  | TROVE2 | chr1 | 193028551 |
| FBXL7 | chr5 | 15500304 |  | | OR2T1 | chr1 | 248569295 |  | TSACC | chr1 | 156307104 |
| FBXO28 | chr1 | 224301788 |  | | OR2T10 | chr1 | 248756130 |  | TSEN15 | chr1 | 184020784 |
| FCAMR | chr1 | 207131311 |  | | OR2T11 | chr1 | 248789478 |  | TSNAX | chr1 | 231664398 |
| FCER1A | chr1 | 159259503 |  | | OR2T12 | chr1 | 248457917 |  | TSTD1 | chr1 | 161007421 |
| FCER1G | chr1 | 161185086 |  | | OR2T2 | chr1 | 248616098 |  | TTC13 | chr1 | 231041986 |
| FCGR1A | chr1 | 149754249 |  | | OR2T27 | chr1 | 248813231 |  | TTC24 | chr1 | 156549518 |
| FCGR1C | chr1 | 149369293 |  | | OR2T29 | chr1 | 248721844 |  | TUFT1 | chr1 | 151512780 |
| FCGR2A | chr1 | 161475204 |  | | OR2T3 | chr1 | 248636651 |  | TXNIP | chr1 | 145438437 |
| FCGR2B | chr1 | 161632904 |  | | OR2T33 | chr1 | 248436153 |  | UAP1 | chr1 | 162531295 |
| FCGR2C | chr1 | 161551128 |  | | OR2T34 | chr1 | 248737101 |  | UBAP2L | chr1 | 154192647 |
| FCGR3A | chr1 | 161511550 |  | | OR2T35 | chr1 | 248801587 |  | UBE2Q1 | chr1 | 154521050 |
| FCGR3B | chr1 | 161592987 |  | | OR2T4 | chr1 | 248524882 |  | UBE2QL1 | chr5 | 6448735 |
| FCRL1 | chr1 | 157764193 |  | | OR2T5 | chr1 | 248651889 |  | UBE2T | chr1 | 202300784 |
| FCRL2 | chr1 | 157715522 |  | | OR2T6 | chr1 | 248550909 |  | UBQLN4 | chr1 | 156005091 |
| FCRL3 | chr1 | 157647977 |  | | OR2T8 | chr1 | 248084319 |  | UCHL5 | chr1 | 192981495 |
| FCRL4 | chr1 | 157543538 |  | | OR2W3 | chr1 | 248058888 |  | UCK2 | chr1 | 165796731 |
| FCRL5 | chr1 | 157483166 |  | | OR2W5 | chr1 | 247654369 |  | UFC1 | chr1 | 161123533 |
| FCRL6 | chr1 | 159770300 |  | | OR6F1 | chr1 | 247875130 |  | UHMK1 | chr1 | 162466963 |
| FCRLA | chr1 | 161676761 |  | | OR6K2 | chr1 | 158669467 |  | URB2 | chr1 | 229761980 |
| FCRLB | chr1 | 161691333 |  | | OR6K3 | chr1 | 158686957 |  | USF1 | chr1 | 161009040 |
| FDPS | chr1 | 155278538 |  | | OR6K6 | chr1 | 158724605 |  | USH2A | chr1 | 215796235 |
| FH | chr1 | 241660856 |  | | OR6N1 | chr1 | 158735533 |  | USP21 | chr1 | 161129253 |
| FLAD1 | chr1 | 154955769 |  | | OR6N2 | chr1 | 158746471 |  | VAMP4 | chr1 | 171669295 |
| FLG | chr1 | 152274650 |  | | OR6P1 | chr1 | 158532440 |  | VANGL2 | chr1 | 160370363 |
| FLG2 | chr1 | 152321212 |  | | OR6Y1 | chr1 | 158516917 |  | VASH2 | chr1 | 213123861 |
| FLVCR1 | chr1 | 213031596 |  | | OTUD7B | chr1 | 149912228 |  | VHLL | chr1 | 156268414 |
| FMN2 | chr1 | 240255184 |  | | OTULIN | chr5 | 14664782 |  | VN1R5 | chr1 | 247419373 |
| FMO1 | chr1 | 171217609 |  | | PAPD7 | chr5 | 6714717 |  | VPS45 | chr1 | 150039349 |
| FMO2 | chr1 | 171154346 |  | | PAPPA2 | chr1 | 176432306 |  | VPS72 | chr1 | 151148775 |
| FMO3 | chr1 | 171060017 |  | | PAQR6 | chr1 | 156213111 |  | VSIG8 | chr1 | 159824105 |
| FMO5 | chr1 | 146655883 |  | | PARP1 | chr1 | 226548391 |  | WDR26 | chr1 | 224572844 |
| FMO6P | chr1 | 171106878 |  | | PBX1 | chr1 | 164528596 |  | WDR64 | chr1 | 241815579 |
| FMOD | chr1 | 203309748 |  | | PBXIP1 | chr1 | 154916558 |  | WNT3A | chr1 | 228194722 |
| G0S2 | chr1 | 209848669 |  | | PCNXL2 | chr1 | 233119881 |  | WNT9A | chr1 | 228109164 |
| GABPB2 | chr1 | 151043079 |  | | PCP4L1 | chr1 | 161228516 |  | XCL1 | chr1 | 168545710 |
| GALNT2 | chr1 | 230193535 |  | | PDC | chr1 | 186412697 |  | XCL2 | chr1 | 168510002 |
| GATAD2B | chr1 | 153777202 |  | | PDCD6 | chr5 | 271735 |  | XPR1 | chr1 | 180601145 |
| GBA | chr1 | 155204238 |  | | PDE4DIP | chr1 | 144851423 |  | YOD1 | chr1 | 207217193 |
| GCSAML | chr1 | 247670359 |  | | PDZK1 | chr1 | 145727665 |  | YY1AP1 | chr1 | 155629232 |
| GGPS1 | chr1 | 235491752 |  | | PEA15 | chr1 | 160175108 |  | ZBED6 | chr1 | 203766650 |
| GJA5 | chr1 | 147228331 |  | | PEAR1 | chr1 | 156863522 |  | ZBTB18 | chr1 | 244212240 |
| GJA8 | chr1 | 147374945 |  | | PEX11B | chr1 | 145516164 |  | ZBTB37 | chr1 | 173837492 |
| GJC2 | chr1 | 228337414 |  | | PEX19 | chr1 | 160246598 |  | ZBTB41 | chr1 | 197122813 |
| GLRX2 | chr1 | 193065594 |  | | PFDN2 | chr1 | 161070345 |  | ZBTB7B | chr1 | 154975105 |
| GLUL | chr1 | 182350838 |  | | PFKFB2 | chr1 | 207226619 |  | ZC3H11A | chr1 | 203764750 |
| GNG4 | chr1 | 235710984 |  | | PGBD2 | chr1 | 249200441 |  | ZDHHC11 | chr5 | 795719 |
| GNPAT | chr1 | 231376918 |  | | PGBD5 | chr1 | 230457391 |  | ZNF124 | chr1 | 247285276 |
| GNRHR2 | chr1 | 145509751 |  | | PGLYRP3 | chr1 | 153270337 |  | ZNF281 | chr1 | 200374074 |
| GOLPH3L | chr1 | 150618700 |  | | PGLYRP4 | chr1 | 153302596 |  | ZNF496 | chr1 | 247463621 |
| GOLT1A | chr1 | 204167287 |  | | PHLDA3 | chr1 | 201434606 |  | ZNF648 | chr1 | 182023704 |
| GON4L | chr1 | 155719448 |  | | PI4KB | chr1 | 151264272 |  | ZNF669 | chr1 | 247263263 |
| GORAB | chr1 | 170501262 |  | | PIAS3 | chr1 | 145575987 |  | ZNF670 | chr1 | 247197939 |
| GPA33 | chr1 | 167022081 |  | | PIGC | chr1 | 172410596 |  | ZNF672 | chr1 | 249132529 |
|  |  |  |  | |  |  |  |  | ZNF678 | chr1 | 227751219 |
|  |  |  |  | |  |  |  |  | ZNF687 | chr1 | 151254790 |
|  |  |  |  | |  |  |  |  | ZNF692 | chr1 | 249144202 |
|  |  |  |  | |  |  |  |  | ZNF695 | chr1 | 247108848 |
|  |  |  |  | |  |  |  |  | ZP4 | chr1 | 238041163 |

**GENES REGIONS IN LOSS REGIONS IN DM vs controls (n=995)**

| **GENES IN LOSS REGIONS IN DM vs controls (n=217)** | | | | | | | |  | |  |  |
| --- | --- | --- | --- | --- | --- | --- | --- | --- | --- | --- | --- |
| Gene_symbol | Chrom | Start |  | Gene_symbol | Chrom | Start |  | | Gene_symbol | Chrom | Start |
| AAED1 | chr9 | 99403532 |  | GNA14 | chr9 | 80037994 |  | | PTGR1 | chr9 | 114312001 |
| ABCA1 | chr9 | 107543283 |  | GNAQ | chr9 | 80331189 |  | | PTPDC1 | chr9 | 96793075 |
| ABHD17B | chr9 | 74477367 |  | GNG10 | chr9 | 114423850 |  | | PTPN3 | chr9 | 112137973 |
| ACTL7A | chr9 | 111624507 |  | GOLM1 | chr9 | 88641057 |  | | RAD23B | chr9 | 110045516 |
| ACTL7B | chr9 | 111616868 |  | GRIN3A | chr9 | 104331633 |  | | RASEF | chr9 | 85594499 |
| AGTPBP1 | chr9 | 88161453 |  | H1F0 | chr22 | 38201113 |  | | RFK | chr9 | 79000432 |
| AKAP2 | chr9 | 112810877 |  | HABP4 | chr9 | 99212436 |  | | RGS3 | chr9 | 116207008 |
| AKNA | chr9 | 117096432 |  | HDHD3 | chr9 | 116135697 |  | | RMI1 | chr9 | 86595636 |
| ALAD | chr9 | 116148591 |  | HIATL2 | chr9 | 99708326 |  | | RNF183 | chr9 | 116059372 |
| ALDH1A1 | chr9 | 75515577 |  | HNRNPK | chr9 | 86582997 |  | | RNF20 | chr9 | 104296130 |
| ALDOB | chr9 | 104182841 |  | HSD17B3 | chr9 | 98997588 |  | | RORB | chr9 | 77112251 |
| AMBP | chr9 | 116822407 |  | HSDL2 | chr9 | 115142188 |  | | S1PR3 | chr9 | 91606323 |
| ANKRD54 | chr22 | 38226861 |  | IDNK | chr9 | 86237963 |  | | SH3BP1 | chr22 | 38035683 |
| ANXA1 | chr9 | 75766780 |  | IKBKAP | chr9 | 111629799 |  | | SHC3 | chr9 | 91620685 |
| ASTN2 | chr9 | 119187503 |  | INIP | chr9 | 115448790 |  | | SLC16A8 | chr22 | 38474143 |
| BAAT | chr9 | 104122698 |  | INVS | chr9 | 102861501 |  | | SLC28A3 | chr9 | 86890764 |
| BAIAP2L2 | chr22 | 38480895 |  | ISCA1 | chr9 | 88879462 |  | | SLC31A1 | chr9 | 115983807 |
| BARX1 | chr9 | 96713908 |  | KIAA0368 | chr9 | 114122972 |  | | SLC31A2 | chr9 | 115913237 |
| BRINP1 | chr9 | 121928907 |  | KIAA1958 | chr9 | 115249247 |  | | SLC35D2 | chr9 | 99075718 |
| BSPRY | chr9 | 116111811 |  | KIF12 | chr9 | 116853917 |  | | SLC44A1 | chr9 | 108006893 |
| C22orf23 | chr22 | 38339056 |  | KIF27 | chr9 | 86451614 |  | | SLC46A2 | chr9 | 115641199 |
| C9orf129 | chr9 | 96080480 |  | KLF4 | chr9 | 110247132 |  | | SMC2 | chr9 | 106856540 |
| C9orf152 | chr9 | 112961845 |  | LGALS1 | chr22 | 38071612 |  | | SNX30 | chr9 | 115513133 |
| C9orf153 | chr9 | 88835179 |  | LINC00474 | chr9 | 118650543 |  | | SOX10 | chr22 | 38368318 |
| C9orf170 | chr9 | 89763558 |  | LINC00476 | chr9 | 98568369 |  | | SPATA31C1 | chr9 | 90532876 |
| C9orf40 | chr9 | 77561498 |  | LINC00587 | chr9 | 105281918 |  | | SPATA31C2 | chr9 | 90744219 |
| C9orf41 | chr9 | 77597872 |  | LOC158434 | chr9 | 98868942 |  | | SPATA31D1 | chr9 | 84603686 |
| C9orf43 | chr9 | 116172923 |  | LPAR1 | chr9 | 113636053 |  | | SPATA31D3 | chr9 | 84558414 |
| C9orf47 | chr9 | 91605777 |  | LPPR1 | chr9 | 103791030 |  | | SPATA31D4 | chr9 | 84543342 |
| C9orf57 | chr9 | 74666296 |  | LRRC37A5P | chr9 | 114365110 |  | | SPATA31E1 | chr9 | 90497771 |
| C9orf64 | chr9 | 86553226 |  | MICALL1 | chr22 | 38302154 |  | | SPIN1 | chr9 | 91003296 |
| C9orf84 | chr9 | 114448798 |  | MRPL50 | chr9 | 104152248 |  | | STX17 | chr9 | 102668914 |
| C9orf85 | chr9 | 74526422 |  | MSANTD3 | chr9 | 103189494 |  | | SUSD1 | chr9 | 114803060 |
| CACNA1B | chr9 | 140772240 |  | MSANTD3-TMEFF1 | chr9 | 103204187 |  | | SVEP1 | chr9 | 113127528 |
| CCDC180 | chr9 | 100069909 |  | MURC | chr9 | 103340360 |  | | TAL2 | chr9 | 108424737 |
| CDC14B | chr9 | 99262394 |  | MUSK | chr9 | 113431050 |  | | TDRD7 | chr9 | 100174301 |
| CDC26 | chr9 | 116029289 |  | NAA35 | chr9 | 88556056 |  | | TEX10 | chr9 | 103064356 |
| CDK20 | chr9 | 90581358 |  | NCBP1 | chr9 | 100395704 |  | | TLE1 | chr9 | 84198597 |
| CEP78 | chr9 | 80850990 |  | NIPSNAP3A | chr9 | 107509968 |  | | TLE4 | chr9 | 82186687 |
| COL27A1 | chr9 | 116917824 |  | NIPSNAP3B | chr9 | 107526450 |  | | TLR4 | chr9 | 120466452 |
| CTNNAL1 | chr9 | 111704848 |  | NMRK1 | chr9 | 77676115 |  | | TMC1 | chr9 | 75136716 |
| CTSL | chr9 | 90340973 |  | NOL12 | chr22 | 38082343 |  | | TMEFF1 | chr9 | 103235519 |
| CTSL3P | chr9 | 90387829 |  | NTRK2 | chr9 | 87283372 |  | | TMEM2 | chr9 | 74298281 |
| CTSV | chr9 | 99791958 |  | NUTM2G | chr9 | 99691285 |  | | TMEM245 | chr9 | 111777414 |
| CYLC2 | chr9 | 105757592 |  | NXNL2 | chr9 | 91150015 |  | | TMEM246 | chr9 | 104237607 |
| DAPK1 | chr9 | 90112142 |  | OR13C2 | chr9 | 107366951 |  | | TMEM38B | chr9 | 108456805 |
| DFNB31 | chr9 | 117164359 |  | OR13C3 | chr9 | 107298050 |  | | TMOD1 | chr9 | 100263461 |
| DNAJC25 | chr9 | 114393631 |  | OR13C4 | chr9 | 107288533 |  | | TRIM32 | chr9 | 119449580 |
| DNAJC25-GNG10 | chr9 | 114393631 |  | OR13C5 | chr9 | 107360737 |  | | TRIOBP | chr22 | 38092994 |
| EHMT1 | chr9 | 140513443 |  | OR13C8 | chr9 | 107331448 |  | | TRPM6 | chr9 | 77337410 |
| EIF3L | chr22 | 38245378 |  | OR13C9 | chr9 | 107379528 |  | | TSTD2 | chr9 | 100362361 |
| EPB41L4B | chr9 | 111934253 |  | OR13D1 | chr9 | 107456702 |  | | TXN | chr9 | 113006091 |
| ERCC6L2 | chr9 | 98637899 |  | OR13F1 | chr9 | 107266543 |  | | TXNDC8 | chr9 | 113065866 |
| ERP44 | chr9 | 102741462 |  | OR2K2 | chr9 | 114089762 |  | | UBQLN1 | chr9 | 86274877 |
| FAM120A | chr9 | 96213977 |  | ORM1 | chr9 | 117085302 |  | | UGCG | chr9 | 114659205 |
| FAM120AOS | chr9 | 96208781 |  | ORM2 | chr9 | 117092068 |  | | VPREB1 | chr22 | 22599199 |
| FAM157B | chr9 | 141106636 |  | OSTF1 | chr9 | 77703397 |  | | VPS13A | chr9 | 79792360 |
| FAM206A | chr9 | 111696672 |  | PALM2 | chr9 | 112403067 |  | | WDR31 | chr9 | 116077930 |
| FANCC | chr9 | 97861335 |  | PALM2-AKAP2 | chr9 | 112542576 |  | | WNK2 | chr9 | 95947211 |
| FKBP15 | chr9 | 115927799 |  | PAPPA | chr9 | 118916070 |  | | ZCCHC6 | chr9 | 88902647 |
| FKTN | chr9 | 108320410 |  | PAPPA-AS1 | chr9 | 119160436 |  | | ZFAND5 | chr9 | 74966340 |
| FOXB2 | chr9 | 79634570 |  | PCSK5 | chr9 | 78505559 |  | | ZFP37 | chr9 | 115804094 |
| FRMD3 | chr9 | 85857904 |  | PDXP | chr22 | 38054736 |  | | ZNF189 | chr9 | 104161135 |
| FRRS1L | chr9 | 111899580 |  | PHF2 | chr9 | 96338908 |  | | ZNF280B | chr22 | 22838771 |
| FSD1L | chr9 | 108210314 |  | PICK1 | chr22 | 38453261 |  | | ZNF367 | chr9 | 99148224 |
| GADD45G | chr9 | 92219926 |  | POLE3 | chr9 | 116169517 |  | | ZNF462 | chr9 | 109625377 |
| GALR3 | chr22 | 38219388 |  | POLR2F | chr22 | 38349669 |  | | ZNF483 | chr9 | 114287438 |
| GAS1 | chr9 | 89559276 |  | PPP3R2 | chr9 | 104353896 |  | | ZNF510 | chr9 | 99518146 |
| GCAT | chr22 | 38203911 |  | PRPF4 | chr9 | 116037913 |  | | ZNF618 | chr9 | 116638561 |
| GCNT1 | chr9 | 79056581 |  | PRUNE2 | chr9 | 79226291 |  | | ZNF782 | chr9 | 99579272 |
| GDA | chr9 | 74729510 |  | PSAT1 | chr9 | 80911990 |  | | ZNF883 | chr9 | 115759399 |
| GGA1 | chr22 | 38004480 |  | PTBP3 | chr9 | 114979994 |  | |  |  |  |
| GKAP1 | chr9 | 86354335 |  | PTCH1 | chr9 | 98205263 |  | |  |  |  |
